# Pressure sensing through Piezo channels controls whether cells migrate with blebs or pseudopods

**DOI:** 10.1073/pnas.1905730117

**Published:** 2020-01-21

**Authors:** Nishit Srivastava, David Traynor, Matthieu Piel, Alexandre J. Kabla, Robert R. Kay

**Affiliations:** ^a^Department of Engineering, University of Cambridge, Cambridge CB2 1PZ, United Kingdom;; ^b^Laboratory of Molecular Biology, Medical Research Council, Cambridge CB20QH, United Kingdom;; ^c^Institut Curie, Université Paris Sciences et Lettres, CNRS, UMR 144, 75005 Paris, France;; ^d^Institut Pierre-Gilles de Gennes, Université Paris Sciences et Lettres, 75005 Paris, France

**Keywords:** cell migration, chemotaxis, blebbing, *Dictyostelium*, Piezo

## Abstract

Blebs and pseudopods can both power cell migration, with blebs often favored in tissues, where cells encounter increased mechanical resistance. To investigate how migrating cells detect and respond to mechanical forces, we used a “cell squasher” to apply uniaxial pressure to *Dictyostelium* cells chemotaxing under soft agarose. As little as 100 Pa causes a rapid (<10 s), sustained shift to movement with blebs rather than pseudopods. Cells are flattened under load and lose volume; the actin cytoskeleton is reorganized, with myosin II recruited to the cortex, which may pressurize the cytoplasm for blebbing. The transition to bleb-driven motility requires extracellular calcium and is accompanied by increased cytosolic calcium. It is largely abrogated in cells lacking the Piezo stretch-operated channel; under load, these cells persist in using pseudopods and chemotax poorly. We propose that migrating cells sense pressure through Piezo, which mediates calcium influx, directing movement with blebs instead of pseudopods.

## Significance

Cells migrating within the body perform vital functions in development and for defense and repair of tissues. In this dense environment, cells encounter mechanical forces and constraints not experienced when moving under buffer, and, accordingly, many change how they move. We find that gentle squashing, which mimics mechanical resistance, causes cells to move using blebs—a form of projection driven by fluid pressure—rather than pseudopods. This behavior depends on the Piezo stretch-operated ion channel in the cell membrane and calcium fluxes into the cell. Piezo is highly conserved and is required for light touch sensation; this work extends its functions into migrating cells.

Cell movement is key to how animals shape their body during embryonic development and defend and repair it as adults (1, 2). In the body, motile cells have to navigate through complex three-dimensional (3D) environments to perform their functions. Unlike the open conditions where movement is often studied, these cells encounter mechanical challenges, such as obstacles, narrow spaces, barrier membranes, and resistance from the extracellular matrix (3, 4). As well as being guided by chemotactic and other cues, cells also need to sense their physical environment, and respond to it appropriately (5–7).

The actin cytoskeleton can drive extension of the cell either by actin polymerization at the leading edge, leading to the formation of pseudopods and similar structures (8–10), or by myosin-driven contraction of the cell cortex, which pressurizes the cytoplasm and favors the formation of blebs (11–13). A key response of cells to tissue-like environments is to favor myosin contractility to drive movement, as in the case of tumor cells in a 3D matrix (14–17). How this change in behavior is triggered is not clear.

Mechanical forces can be sensed by the actin cytoskeleton itself, which intrinsically adapts to load (18, 19), or by stretchable proteins acting as strain gauges (20, 21), or by stretch-operated channels in the plasma membrane (22, 23). Most relevant here is the Piezo channel, which is opened by strain in the membrane and lets through a variety of cations, including calcium (24–26); it is responsible for touch sensation, stem cell differentiation, and sensing of crowding in epithelia among many other things (27–30), but there is only limited evidence for a role in mechanical sensing during cell migration (31).

The very complexity of natural cellular environments makes it hard to tease out how such changes in cell behavior are triggered (32). If it is purely a mechanical response, what are the nature and magnitude of the forces that cells detect, how are they are detected, and what is the response pathway? Simplified systems are useful to analyze this complexity.

*Dictyostelium* amoebae move through varied environments during their life cycle. As single cells, they hunt bacteria through the interstices of the soil, and when starved and developing, they chemotax to cyclic AMP and move in coordinated groups that develop into stalked fruiting bodies, with cell sorting playing a key role (33, 34). We found previously that *Dictyostelium* cells prefer pseudopods when moving under buffer, but blebs under a stiff agarose overlay (35). In both cases, the cells move on the same glass substratum, but under agarose they must also break adhesive forces between the substratum and the overlay and they experience elastic forces caused by deforming the overlay itself. The cells therefore experience both increased mechanical resistance at the leading edge and compression of the cell body. It seems likely that one or both of these somehow trigger the switch to bleb-driven movement.

In order to study how mechanical forces trigger a change in movement mechanics, we built a “cell squasher” to rapidly apply defined loads to cells under an agarose overlay (36) while leaving other potential variables, such as chemical composition and degree of cross-linking of the matrix, or even oxygen availability, largely constant. Using *Dictyostelium* cells, this has allowed us to investigate one variable—the uniaxial load on cells—in isolation. We find that modest loads rapidly cause cells to switch to a bleb-driven mode of movement and that this depends almost entirely on the Piezo stretch-operated channel, most likely acting through a calcium signal to reconfigure the motile apparatus toward myosin II-driven contractility.

## Results

### Uniaxial Loads Cause Cells to Rapidly Adopt Bleb-Driven Motility.

**Fig. 1.**
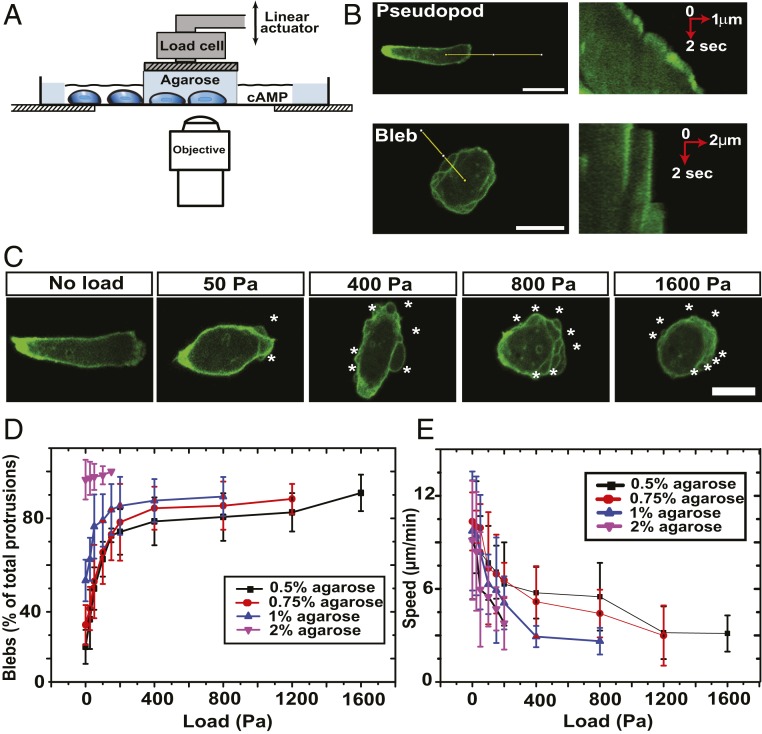
Uniaxial load causes cells to move using blebs instead of pseudopods. (*A*) Diagram of the cell squasher used to apply uniaxial loads to cells moving under an agarose overlay (36). The load is applied using a plunger on the bridge between two wells cut into the agarose, one containing cells and the other, the chemoattractant cyclic AMP, which attracts the cells under the agarose toward it. (*B*) Distinction between pseudopods and blebs. At the *Left* are shown cells expressing an F-actin reporter, and at the *Right*, kymographs taken along the lines indicated at the *Left*. (Scale bar: 10 µm.) (*C*) Uniaxial load causes cells to migrate using blebs. The cells are migrating under an overlay of 0.5% agarose to which increasing uniaxial loads are applied. Blebs are indicated by white stars. (Scale bar: 10 µm.) (*D*) Blebbing of migrating cells increases with increasing load or overlay stiffness. (*E*) Cell speed decreases under increasing load or stiffness of the agarose overlay. The data are represented as mean ± SD for *n* ≥ 30 cells for each case with measurements made for about 30 min, starting 8 to 10 min after load was applied. The stiffness of the agarose overlays is as follows: 0.5% = 6.6 kPa; 0.75% = 11.9 kPa; 1% = 20.5 kPa; and 2% = 73.6 kPa. Aggregation-competent Ax2 cells expressing the ABD120-GFP reporter for F-actin and migrating toward cyclic AMP in KK2MC are used throughout.

We used a custom-built cell squasher to apply uniaxial loads to *Dictyostelium* cells moving under a thin layer of agarose on a glass surface (36) (Fig. 1*A*). In most experiments, cells were chemotaxing to cyclic AMP and transformed with fluorescent reporters. Blebs and pseudopods were distinguished by their characteristic morphologies and dynamics, as revealed by an F-actin reporter (35) (Fig. 1*B*). Blebs are rounded, expand abruptly, and leave behind an F-actin scar (the former cell cortex), which dissipates over a few tens of seconds. Their membrane is initially free of F-actin but rapidly reforms an F-actin cortex. In contrast, pseudopods are more irregular, expand steadily but more slowly, and always have F-actin at their leading edge.

We first showed that mechanical load alone is sufficient to cause cells to switch to migrating predominately with blebs (36). Cells under a soft 0.5% agarose overlay (Young’s modulus, 6.6 kPa) predominantly move with pseudopods, which form ∼75% of large projections (Fig. 1 *C* and *D* and Movie S1). Loads of as little as 50 Pa cause a detectable shift to blebs, which is half-maximal at 100 Pa (Fig. 1 *C* and *D* and Movie S2). At higher percentages of agarose in the overlay, the basal level of blebbing is greater, but load again causes an increase to approaching 100% with 2% agarose (Fig. 1*D*; Young’s modulus, 75 kPa). Cells also move more slowly under load, again proportional to the load applied and the stiffness of the overlay (Movies S3 and S4).

**Fig. 2.**
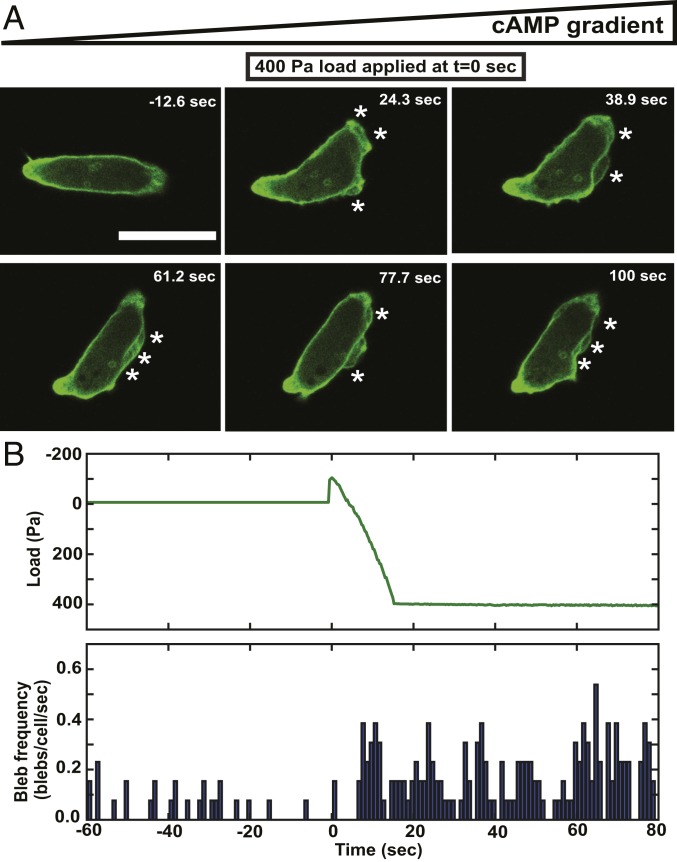
Uniaxial load causes a rapid switch to bleb-driven migration. (*A*) Rapid induction of blebbing by uniaxial loading of migrating cells. Frames from a movie timed with respect to the start of loading (*t* = 0); blebs are indicated by an asterisk (*). (Scale bar: 10 μm.) (*B*) Time course of bleb induction by load. At the *Top* is shown a typical loading regime with a small up-tick as the plunger first touches the agarose followed by a 15-s ramping of load to 400 Pa. At the *Bottom* is shown the bleb frequency, with blebs binned into 1-s intervals and scored at the time they first appear (typically, they are fully expanded in one frame of the movie). Aggregation-competent Ax2 cells expressing the F-actin reporter ABD120-GFP were filmed at 2 frames per s under a 0.5% agarose overlay (*n* = 17 cells).

The switch to bleb-driven motility might result from slow processes such as gene expression changes, which would take many minutes or hours to come into effect (16) or be controlled directly by signal transduction. We therefore asked how quickly cells respond to changes in load. Within 10 s of applying load, there is a clear increase in blebbing from about 2 to 10 blebs per cell per min (Fig. 2 and Movie S5). This may even be an underestimate of how quickly cells respond, since we ramp up the load over 20 s to avoid a loss of focus. The increased rate of blebbing is sustained as long as the load is maintained but can be reversed in 8 to 10 min if it is removed, with cells again moving predominantly with pseudopods (*SI Appendix*, Fig. S1 and Movie S6) and forming only about 3 blebs per cell per min (*SI Appendix*, Fig. S1*B*).

These results show that uniaxial pressure alone is sufficient to make cells move using blebs instead of pseudopods, and that the speed of response is too fast to be due to changes in gene expression: Cells must possess a fast-acting response system to mechanical load.

### Load Makes Cells Shrink and Affects Actin Dynamics.

To understand better the effects of load, we investigated changes in cell morphology and the actin cytoskeleton (*SI Appendix*, Table S1). Cells flatten under load, with a reduction in their height and volume as measured from 3D reconstructions. For instance, a load of 400 Pa applied to cells under 0.5% agarose causes their height to decrease from 7 ± 1 to 3 ± 1 µm (*SI Appendix*, Table S1).

Cell volume, measured from confocal reconstructions, decreased by about 25% under 400 Pa of pressure (*SI Appendix*, Table S1). This was surprising since the volume of freely moving *Dictyostelium* cells is relatively stable, even though their surface area can change by as much as 30% over a few minutes (37). This result was confirmed by two independent methods. In the first, we found that the fluorescent intensity of soluble GFP increases by 45% in confocal sections when load is applied, indicating an increase in GFP concentration and hence a decrease in cytosolic volume. In the second, we used a dye exclusion method to measure the volume of cells constrained in chambers of different heights (38). Although dynamic load could not be applied in this case, we found that the volume of cells constrained to a height of 4 µm was 25% less than cells constrained to 6 µm (*SI Appendix*, Table S1, bottom panel).

Compressive load also causes a loss of polarity. Without load, cells are typically polarized with a pronounced leading edge directed up the cyclic-AMP gradient (Fig. 1*C*). At loads above 100 Pa, as blebs increase, the cells become less polarized, and at higher loads still, they round up and a distinct leading edge is completely lost.

**Fig. 3.**
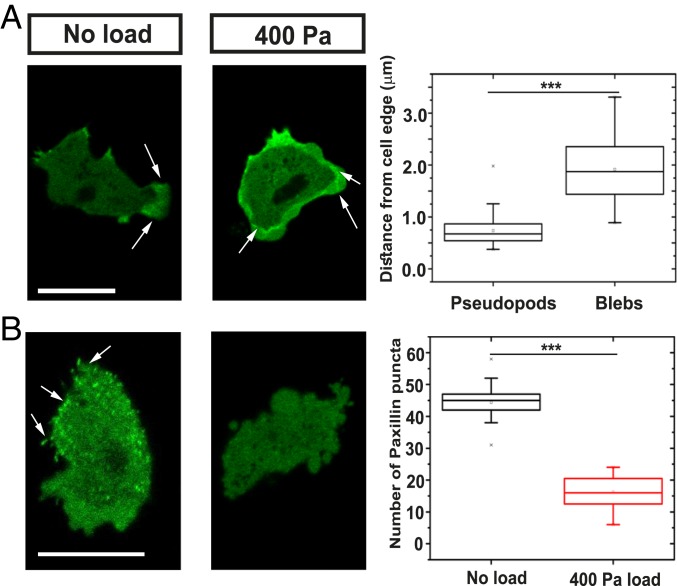
Uniaxial load causes cytoskeletal reorganization. (*A*) Coronin, an F-actin binding protein, relocates under load from pseudopods to the actin scars left behind by blebs. Quantification of the coronin localization from the cell edge. Data are represented as mean ± SD for *n* ≥ 40 cells for each case; one-way ANOVA, ***P < 0.005. (*B*) Paxillin patches, thought to mediate adhesion to the substratum, disperse under load. Quantification of the number of paxillin patches in the cell under different loading conditions. Data are represented as mean ± SD for *n* ≥ 20 cells for each case; one-way ANOVA, ***P < 0.005. Load was applied to aggregation-competent Ax2 cells expressing either coronin–GFP or GFP–paxillin and migrating toward cyclic AMP, under an overlay of 0.5% agarose. (Scale bar: 10 µm.)

The actin cytoskeleton is substantially perturbed under load. Coronin, an F-actin binding protein required for efficient chemotaxis (39, 40), redistributes from pseudopods to the F-actin scars left by expanding blebs (Fig. 3*A*), and these scars tend to linger. Quantitation (*SI Appendix*, Fig. S2 and *Material and Methods*) shows an increase from a half-life of 4 ± 1 s in control cells to 11 ± 1 s under 400 Pa and 13 ± 7 s under 800 Pa load, where the cell perimeter becomes marked by slowly dissipating arcs of F-actin (*SI Appendix*, Table S1). The basal surface of migrating cells is decorated by punctate local adhesions containing paxillin (41), which can be visualized with paxillin–GFP. We find that these tend to dissipate under load, suggesting that cells become less adhesive when moving with blebs (Fig. 3*B*).

### Load Causes Myosin II Recruitment to the Cell Cortex.

**Fig. 4.**
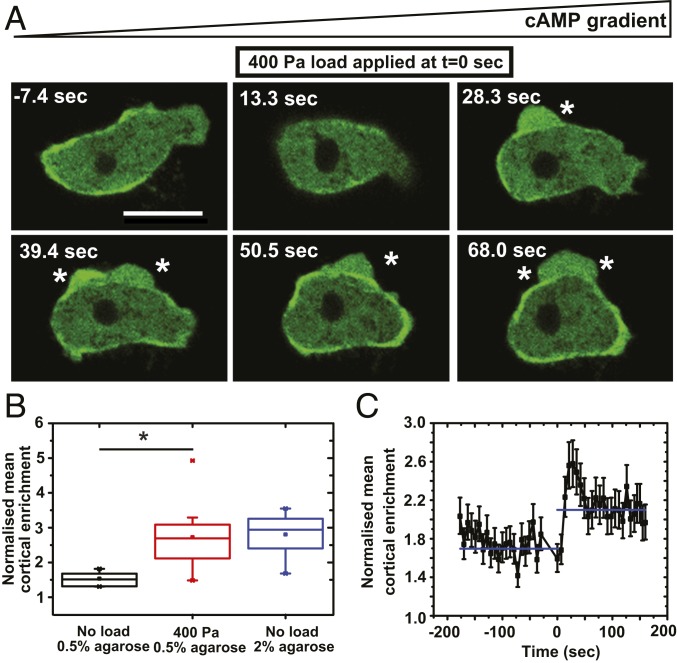
Myosin II is rapidly recruited to the cell cortex in response to load. (*A*) Load causes myosin II to be recruited to the cell cortex. Blebs are indicated by an asterisk (*). (Scale bar: 10 µm.) (*B*) Quantification of cortical enrichment of myosin II under load. Data are represented as mean ± SD for *n* ≥ 20 cells for each case; one-way ANOVA, *P < 0.0005. (*C*) Time course showing that myosin II is rapidly recruited to the cell cortex under load. Data are given as mean ± SEM; *n* = 10 cells; one-way ANOVA, *P* < 0.005. Cortical enrichment is calculated by measuring the ratio of fluorescence intensity of membrane and cytoplasm around the cell. Ax2 cells expressing myosin II–GFP (GFP–MhcA) and chemotaxing to cyclic AMP under 0.5% agarose gels were used throughout. In time courses, load is applied at *t* = 0.

We next investigated the mechanism by which load causes increased blebbing. Blebs are driven out by fluid pressure, which is produced by contraction of the cell cortex driven by myosin II. We confirmed that load-induced blebbing depends on myosin II (*SI Appendix*, Fig. S4*D*), as does blebbing in other circumstances (35, 42). A GFP–myosin II reporter expressed in myosin II-null cells restores their ability to bleb and is therefore functional. This reporter accumulates preferentially in the cortex toward the rear of cells chemotaxing primarily with pseudopods; however, a 400-Pa load causes a sudden and more uniform recruitment to the cortex (Fig. 4*A* and Movie S7), with enrichment over the cytoplasm increasing by about 75% from 1.6 ± 0.2 to 2.8 ± 0.2 (Fig. 4*B* and *SI Appendix*, Fig. S3). Myosin II is recruited in less than 20 s and so is on the same timescale as the increase in blebbing (Fig. 4*C*).

The spatial distribution of myosin II can be used as a polarity marker and quantified by Fourier analysis (*SI Appendix*, Fig. S3 and *Materials and Methods*). Its distribution changes from 0.2 ± 0.04 AU without load to 0.1 ± 0.05 AU under 400-Pa load (*SI Appendix*, Fig. S4*A*). This transition to a more uniform distribution under load occurs on a similar timescale as recruitment to the cortex (*SI Appendix*, Fig. S4*B*) and confirms the visual impression that cells lose polarity when load is applied. Increased cortical accumulation of myosin is also a feature of cells under stiffer gels (Fig. 4*B* and *SI Appendix*, Fig. S4*C*), but in this case myosin II accumulates preferentially at the rear, unlike the more uniform recruitment in squashed cells.

Collectively, the results in the last two sections show that as well as inducing blebbing, load makes cells shrink and causes a profound reorganization of the actin cytoskeleton, including persistent recruitment of myosin II to the cortex. Here, it likely increases contractility, pressurizing the cytoplasm to favor blebbing.

### Calcium Signaling May Mediate the Response to Load.

**Fig. 5.**
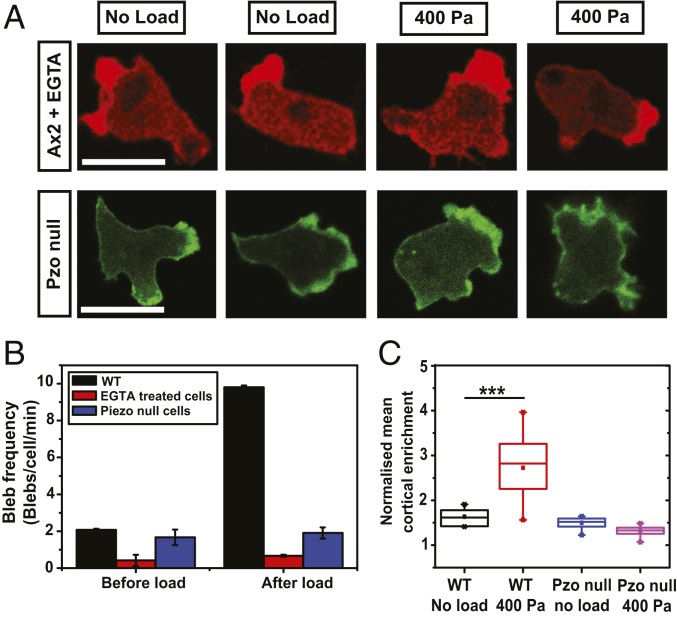
Extracellular calcium and the Piezo stretch-operated channel are required for cells to respond to load. (*A*) Illustration of typical responses to load of cells either in calcium-free medium, or lacking the Piezo channel (PzoA^−^ cells, strain HM1812). Compare to Ax2 controls in Figs. 1*C* and 2*A*. (Scale bar: 10 µm.) (*B*) Quantification of the blebbing response to load of cells either in calcium-free medium, or lacking the Piezo channel. Data are mean ± SD for *n* ≥ 15 cells tracked before and after applying load in each case. Cells, either Ax2 parental or Piezo-null mutant (PzoA^−^, strain HM1812), were incubated under agarose made with the standard KK2MC buffer, which has 100 µM calcium, or this buffer lacking calcium and supplemented with 200 μM EGTA. A load of 400 Pa was applied as indicated. (*C*) Quantification of the cortical enrichment of myosin II in PzoA^−^ cells under a load of 400 Pa. The cortical enrichment of RFP–myosin II is calculated by measuring the ratio of fluorescence intensity of membrane and cytoplasm around the cell. Cells are chemotaxing to cyclic AMP under 0.5% agarose gels. The data are mean ± SD for *n* ≥ 20 cells analyzed for each case; ***P < 0.0005 for wild-type cells and *P* > 0.5 for Piezo-null cells, Mann–Whitney *U* test and one-way ANOVA.

We hypothesized that cells possess a dedicated mechanosensing system for their response to load. One possible route is through an influx of calcium into the cytoplasm, mediated by stretch-operated channels. We tested whether the response to load depends on external calcium by using a nominally calcium-free medium, with 200 μM EGTA included to chelate any traces remaining. In this medium, cells continue to move and produce a basal number of blebs (although reduced), but the increase caused by load is virtually abolished (Fig. 5*A* and Movie S8). In control cells, blebs increase from 2.1 ± 0.1 to 9.1 ± 0.1 blebs per cell per min when a load of 400 Pa is applied, whereas in those treated with EGTA, they only increased from 0.4 ± 0.3 to 0.9 ± 0.1 blebs per cell per min (Fig. 5*B*). Instead, the cells move predominantly with pseudopods, which constituted more than 90% of the projections produced under a steady-state load of 400 Pa.

**Fig. 6.**
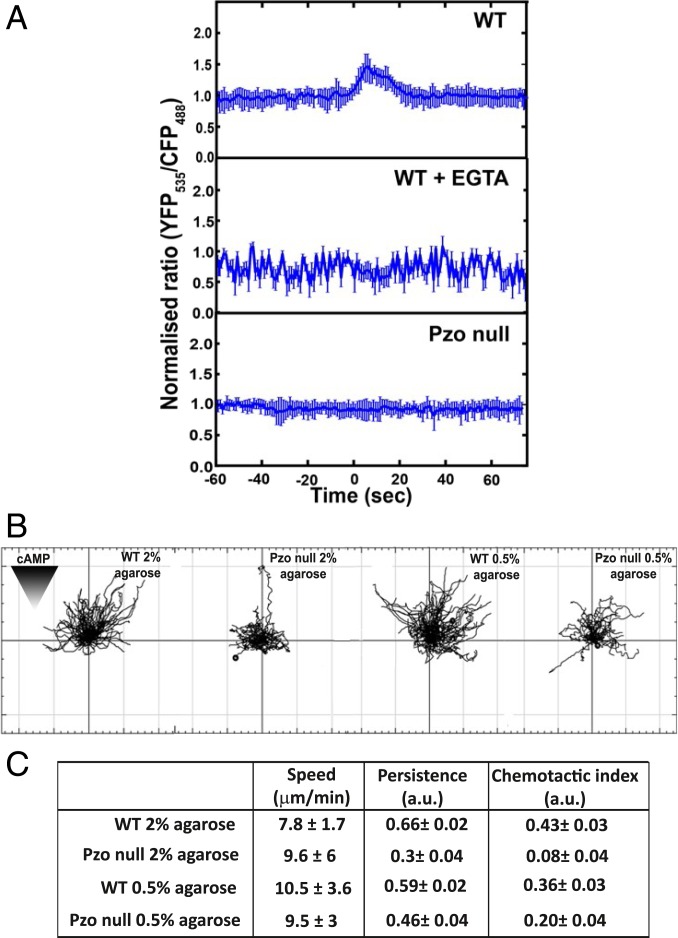
The Piezo channel is required for the calcium response to load and for efficient chemotaxis. (*A*) Loading causes a transient increase in cytosolic calcium, which depends on extracellular calcium and the Piezo channel. Changes in cytoplasmic calcium were detected by microscopy using the Cameleon FRET-based sensor. The normalized ratio of YFP (535 nm)/CFP (485 nm) indicates the cytosolic calcium concentration. Aggregation-competent cells under 0.5% agarose were subjected to a load of 400 Pa as indicated (*n* = 15 cells). (*B*) Piezo-null cells are defective in chemotaxis to cyclic AMP when constrained under agarose. Representative tracks of wild-type (WT) (strain Ax2, R.R.K. laboratory) and Piezo-null cells (PzoA^−^ cells, strain HM1812) chemotaxing toward a cyclic-AMP source in an under-agarose assay. Agarose overlays of different stiffness (0.5% and 2% agarose with Young’s modulus of 6.6 and 73.6 kPa, respectively) were used. (*C*) Table of the chemotactic parameters obtained by tracking cells in an under-agarose assay. Speed was calculated by dividing the accumulated distance by total time. Persistence is defined as the ratio between Euclidian distance and accumulated distance, and chemotactic index is defined as the cosine of the angle between net distance traveled in the direction of the gradient and the Euclidian distance. Data are represented as mean ± SEM from measurements obtained for *n* ≥ 50 cells on at least three different days; *P* < 0.005, Mann–Whitney *U* test and one-way ANOVA.

To ask whether loading a cell causes an increase in cytoplasmic calcium, we used cells expressing the YC2.60 fluorescence resonance energy transfer (FRET) reporter for calcium (43). The results show that load causes an immediate, although modest, increase in the normalized FRET ratio: It increases from an average value of 1.0 before load to a maximal of 1.6 shortly after load is applied, returning to baseline of 1.1 in about 20 to 25 s (Fig. 6 *A*, *Top*). The response is clear but much smaller than that to a saturating dose of 4 μM cyclic AMP or 4 μM ionomycin (*SI Appendix*, Fig. S5*A*). In the presence of EGTA, the normalized FRET ratio hovered around 0.8 with no appreciable increase on loading (Fig. 6 *A*, *Middle*), indicating that the increase in cytosolic calcium depends on an influx through the plasma membrane.

These results therefore suggest that the load applied to cells is sensed by an influx of calcium into the cytoplasm, mediated by an unknown mechanosensitive channel in the plasma membrane.

### The Piezo Stretch-Operated Channel Is Required for Sensing Load.

Only a limited number of potential stretch-operated channels are recognizable in the *Dictyostelium* genome (44). We screened null mutants in these for defects in response to load. A triple mutant of the mechanosensing channel MscS and two Trp channels (one a mucolipin homolog, MclN; the other, TrpP, which is responsive to ATP) (43) and a double mutant of IplA (a homolog of the IP3 receptor required for the calcium response to chemoattractants) (45), and the TrpP channel, both showed a normal response to load, making essentially wild-type numbers of blebs (*SI Appendix*, Fig. S6 and Movies S9–S12).

In addition to these channels, the *Dictyostelium* genome encodes a single homolog of the Piezo stretch-operated channel [DDB_G0282801 at dictyBase (46); we designate the gene as *pzoA*]. We created knockout mutants in this gene (*SI Appendix*, Fig. S7), which grew close to normally in shaken suspension in HL5 liquid medium (mean generation times: 8.59 ± 0.11 h for the Ax2 parent and 9.18 ± 0.12 and 9.56 ± 0.13 h for the HM1812 and HM1813 *pzoA*^*−*^ mutant strains; SEM; *n* = 3).

It is immediately apparent that the HM1812 Piezo mutant behaves differently under load from its parent, continuing to move with pseudopods instead of blebs (Fig. 5 *A* and *B*). To confirm this, mutant and parent were marked with different colored fluorescent F-actin reporters, mixed, and subjected to a load of 400 Pa: the parent blebs copiously, as expected, but the mutant barely at all (*SI Appendix*, Fig. S8*A* and Movie S13). Instead, the mutant continues to move with actin-driven pseudopods. A second Piezo mutant—HM1813—behaved similarly (*SI Appendix*, Fig. S9 and Movie S14). Quantification shows that the mutant produces a basal level of blebbing without load, but unlike its parent, load causes little if any increase (Fig. 5*B* and *SI Appendix*, Fig. S8 *B* and *E*). This is true even for a load of 1,600 Pa (*SI Appendix*, Fig. S8*B* and Movie S15). Similarly, inducing the mutant to move under stiffer agarose gives only a small increase in blebbing over basal, whereas blebbing in the parent increases to around 90% of projections under 2% agarose (*SI Appendix*, Fig. S8 *C* and *D*).

Piezo-null cells still produce basal levels of blebs, suggesting that blebbing does not intrinsically depend on Piezo. To test this, we stimulated cells with cyclic AMP, which is detected by cAR1, a G-protein–coupled receptor, and found that it triggers a burst of blebbing in both wild-type (42) and Piezo-null cells (*SI Appendix*, Fig. S5*B* and Movie S16). Thus, the ability of Piezo-null cells to bleb remains intact, but it can no longer be stimulated by mechanical means.

Cytosolic calcium levels in Piezo-null cells were not perceptibly stimulated by load (Fig. 6 *A*, *Bottom*). Although the traces are quite noisy, the normalized FRET ratio fluctuated around 0.8 before the application of load and did not show any appreciable change under a load of 400 Pa. Similarly, load does not cause cortical recruitment of myosin II in Piezo-null cells (Fig. 5*C*, *SI Appendix*, Fig. S10, and Movie S17), and their polarity is unchanged (*SI Appendix*, Fig. S10).

The inability of Piezo-null cells to respond to uniaxial pressure has severe consequences for their movement under confinement. Under a 2% agarose overlay, wild-type Ax2 cells chemotax quite efficiently toward cyclic AMP, predominantly using blebs. Piezo-null cells move at a similar speed using F-actin–driven projections, but chemotax poorly, following tortuous, zig-zag paths with poor directionality (Fig. 6 *B* and *C*). Under 0.5% agarose, the mutant cells are still defective but much more similar to their parent.

## Discussion

Cells need to migrate through mechanically varied terrains to perform their physiological functions, and many can adjust how they move accordingly. We showed previously that *Dictyostelium* amoebae prefer to migrate using pseudopods under buffer but switch to blebs under a stiff agarose overlay (35, 47). The advantage of this change in movement mechanics is unclear, but the behavior resembles that of tumor cells migrating through 3D matrices (14, 15, 32). We show here that, when uniaxial pressure is applied to cells migrating under soft agarose, it also induces them to move using blebs instead of pseudopods (36). The signal to the motility apparatus depends on the Piezo stretch-operated channel and is likely transmitted by an influx of calcium.

**Fig. 7.**
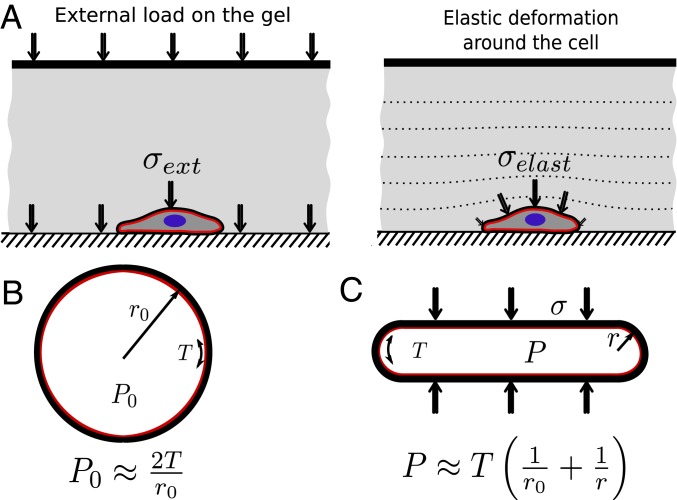
Biophysical representation of the changes in cell geometry and pressure upon application of external load and the proposed link to Piezo activation. (*A*) Illustration of two mechanisms for applying compressive load to a cell. (*Left*) External loading is imposed from the upper boundary and transmitted through the gel and cells to the substrate. (*Right*) Elastic overlays would generate stress when deformed to accommodate a cell between them and a rigid substrate, and so apply load to the cell. (*B*) In a spherical cell, the Laplace equation links the hydrostatic pressure, *P*_0_, in the cytosol, the radius, *r*_0_, of the cell, and its cortical tension *T*. (*C*) Application of external load to a spherical cell leads to its flattening and change to a pancake shape. In this case, the top surface is approximately flat, and the external load *σ* must be balanced by a higher cell hydrostatic pressure *P*. However, the load is not acting along the periphery of the cell; there, Laplace law now relates the internal pressure *P* with cortical tension and increased local curvature, therefore relating external load *σ* to the cell height. We propose the increased cytosolic pressure, resulting from loading and squashing the cell, activates the Piezo channels by increasing tension in the plasma membrane.

Cells migrating under agarose distort the overlay to make room for their body and so experience elastic forces from the overlay in return; in this way, they are similar to cells to which pressure from the squasher is applied (Fig. 7). In both situations, the compressive forces flatten the cells and, when blebbing is maximal, reduce their height by about one-half.

Blebs are expanded by fluid pressure produced by the contractile acto-myosin cortex, with the pressure varying according to the cortical tension and curvature, following the Laplace law. The Laplace pressure for a rounded cell is P_0_ = 200 Pa using a cortical tension of *T* = 0.5 mN/m (48) and assuming a spherical cell of radius *r* = 5 μm. This increases to 300 Pa in a cell flattened to half this height. Thus, squashing cells can easily cause an increase in cytosolic pressure of a comparable order to the “basal” pressure of a rounded cell. We propose that this increased pressure increases tension in the plasma membrane, which is anchored to the cell cortex at discrete attachment points, and that the increased tension opens Piezo channels to let calcium and other ions into the cell. In this way, cells can sense physical forces and change their behavior. The phenomenology also agrees with the blebbing observed in many mammalian cells when they are flattened (6) and is therefore likely to be generic.

Squashing cells causes the loss of at least 25% of their volume, and given the extreme crowding of the cytoplasm, this could have profound effects on properties such as viscosity (49) and processes including actin polymerization and depolymerization (50). Similar changes in cell volume occur when glioma cells invade narrow spaces (51). In contrast to some mammalian cells (6), squashing of the nucleus seems unlikely to play a major role in the responses we observe, because at 2-µm diameter (52), it is still smaller than the height of a squashed cell.

The cytoskeleton is also reorganized by squashing. Myosin II is essential for blebbing (16, 17, 53) and is strongly recruited to the cell cortex, where it is expected to increase contractility and so pressurize the cytoplasm. Coronin is redeployed from pseudopods to the F-actin scars left behind by expanding blebs, which it may help to break down (54). Finally, paxillin is lost from adhesive puncta on the bottom of the cell (41), suggesting that adhesion to the substratum is reduced, similar to other cells, which also become less adhesive when moving in a “pressure-driven” mode (55).

Piezo channels are inherently mechanosensitive and, when opened by membrane tension, allow various cations into the cell, including Ca^2+^ (24). As well as requiring Piezo, the blebbing response to load depends on extracellular Ca^2+^ and is associated with a transient increase in cytosolic Ca^2+^. We therefore propose that it is mediated by an increase in cytosolic Ca^2+^ produced by Piezo channels. It is not clear how this transient Ca^2+^ signal gives a persistent change in cell behavior. Possibly, it acts as a switch that is reversed by some other means when the pressure is removed; or local increases in cytosolic Ca^2+^ persist in the submembranous region of the cytoplasm but are too small to detect by our methods.

Myosin II is also recruited to the cell cortex when cells are stimulated with the chemoattractant cyclic AMP, but this occurs even when extracellular Ca^2+^ is chelated with EGTA and in the IplA mutant, where there is no detectable increase in cytosolic Ca^2+^ (56–58). Nor do myosin heavy chain kinase or myosin light chain kinase appear to be directly regulated by calcium (59). Thus, the link between myosin II recruitment stimulated through Piezo is likely to differ from that caused by chemoattractant.

There is only one Piezo gene in the *Dictyostelium* genome (44). Mutant cells grow normally in liquid medium, and their morphological development is normal when they are starved on buffered agar. Mutant cells can still move under stiff agarose using actin-driven projections. However, their chemotaxis is severely impaired: Instead of moving relatively smoothly up a cyclic-AMP gradient, they follow tortuous paths with poor directionality. Blebs and pseudopods cooperate in chemotaxing cells, with both orientating preferentially up-gradient (35, 47, 60), and it may be that this cooperation is particularly important under severe mechanical restriction.

Piezo is highly conserved and has already been linked to a large number of mechanically sensitive processes (23, 24, 29, 30, 61–64). Following this *Dictyostelium* precedent, we see no reason why many other types of migrating cell should not also sense their mechanical environment through Piezo.

## Materials and Methods

Detailed information about the cell culture, under-agarose and cell-squashing experiments, and image methods can be found in *SI Appendix*. Briefly, *Dictyostelium* cells (strain Ax2; R.R.K. laboratory) were grown axenically in HL5 medium at 22 °C. Migration experiments under agarose gels were performed using aggregation competent cells, prepared by starving logarithmically growing cells in KK2MC buffer and after 1 h, pulsing them with cyclic AMP every 6 min for 4.5 h. A modified under-agarose assay was used in which an agarose gel of 2-mm height was poured in a glass-bottom dish and two rectangular troughs were cut into it, one containing cyclic AMP and the other, cells. Load was applied to the cells once they had chemotaxed underneath the agarose overlay (36). Blebs and pseudopods were scored morphologically as well as using kymographs. Speed of cells was calculated using QUIMP software. Cell height was measured by the reconstruction of *z* stacks while volume was computed by the sum of volume of all of the voxels occupied by a cell. The distribution and localization of myosin were measured using a MATLAB plug-in, which is described in detail in *SI Appendix*. The data are available upon request from the corresponding author.

## Supplementary Material

Supplementary File

Supplementary File

Supplementary File

Supplementary File

Supplementary File

Supplementary File

Supplementary File

Supplementary File

Supplementary File

Supplementary File

Supplementary File

Supplementary File

Supplementary File

Supplementary File

Supplementary File

Supplementary File

Supplementary File

Supplementary File
