# Supplementary material for: Pressure sensing through Piezo channels controls whether cells migrate with blebs or pseudopods
Source: Proc Natl Acad Sci U S A. 2020 Jan 21;117(5):2506–12. doi: 10.1073/pnas.1905730117 (PMC7007555; doi:10.1073/pnas.1905730117)
Supplement: Supplementary File [file pnas.1905730117.sapp.pdf]

Supplementary information for

Pressure sensing through Piezo channels controls whether cells migrate with blebs or pseudopods

Nishit Srivastava[1,3,4], David Traynor[2,5], Matthieu Piel[3,4], Alexandre J. Kabla[1] & Robert R. Kay[2*]

1. Department of Engineering, University of Cambridge, Cambridge CB2 1PZ, UK

2. MRC Laboratory of Molecular Biology, Francis Crick Avenue, Cambridge CB20QH

3. Institut Curie, PSL Research University, CNRS, UMR 144, 75005, Paris, France

4. Institut Pierre-Gilles de Gennes, PSL Research University, 75005, Paris, France

Current Addresses:

3. Institut Curie, PSL Research University, CNRS, UMR 144, F-75005, Paris, France

4. Institut Pierre-Gilles de Gennes, PSL Research University, F-75005, Paris, France

5. Cambridge Institute for Medical Research, Hills Road, Cambridge CB2 0XY

***corresponding author: Robert Kay - rrk@mrc-lmb.cam.ac.uk**

This supplementary document includes:
Detailed materials and methods
Supplementary figure legends (S1 to S9)
Table
Caption for movies (S1 to S16)
Supplementary figures (S1 to S9)

Online Materials and Methods

Cell culture and Reporters

Dictyostelium discoideum, strain Ax2 (Kay laboratory strain; DBS0235521 at http://dictybase.org/) was grown at 22°C on *Klebsiella aerogenes* lawns on SM agar plates (Kay,1987) or axenically in HL5 medium (Formedium). It was transformed with an F-actin reporter, ABD-GFP consisting of the F-actin binding domain of ABP-120 (residues 9-248) fused to GFP driven by the actin15 promoter (1) (strain HM2040). The expression of ABD-GFP was maintained under the selection of 10 μg/ml of G418 antibiotic.

The GFP-MhcA reporter for myosin-II, was created from an extra-chromosomal GFP expression vector (pDM1407) and pJET-MhcA cloning vector (2) and an mCherry-MhcA vector was created similarly using an mCherry expression vector (pDM1208). A fluorescent reporter for Paxillin was created in a similar manner using pDM1407 and a cloning vector for paxillin (pDM722) to create GFP-paxillin.

The Piezo gene homologue (DDB0282801) in *Dictyostelium* was knocked out using the vector pDT43 (Supplementary figure S7A). A 1.56 kb 5'region of flanking homology was generated by PCR and cloned into pLPBLP as a ApaI/HindIII fragment. Then a 1.32kb 3' region of flanking homology was generated by PCR and cloned in as a NotI/SacII fragment to generate pDT43. Primer pairs used for PCR were PZKO1/PZKO2(5') and PZKO3/PZKO4(3').

PZKO1:5'-TTTGGGCCCTAAATTTATCCTTTTTCATTGATTTGTTCATCAG-3'

PZKO2:5'-ATTAAAGCTTATGCTAATAATATCGCTGATGCTACACTTGCTG-3'

PZKO3:5'-TAAGCGGCCGCTTGAAGTCTCTGTTGTAGGTTGGAATCC-3'

PZKO4:5'-TAACCGCGGTAATTATTATGATACAATTGCTGATATGGAAGCTGC-3'

Ax2 cells were electroporated with digested pDT43 (digested with ApaI/SacII) and transformants selected with blasticidin (20 µg/ml). Genomic DNA was prepared from wells using GenElute kit (Qiagen). For PCR screening, the following primers were used:

PZ1: 5'- GGAAATAAAAAAATGATAGGATATTTCTTTGTG -3'and

PZ2: 5'- TGGTAAAACTGTTTCACAAGTTGCTACTTCC -3', which bound outside the disruption cassette. PzoA KO cells gave a product at 4.42 kb and were found in 2/14 wells. These were re-cloned and assessed by PCR (Supplementary figure S7B).

Live-cell microscopy and cell squashing experiments

All microscopy was with cells in KK2MC buffer (16.5 mM KH_2PO_4, 3.8mM K_2HPO_4, 2 mM $MgSO_4$, and 0.1 mM $CaCl_2$, pH ~ 6.2) unless stated otherwise. Freshly starved cells were brought to aggregation-competence in KK2MC at $2x10^7$ cells/ml with shaking at 180 rpm. After 1 hour cells were pulsed with 90 nM final cyclic-AMP, every 6 minutes for another 4.5 hours. Aggregation-competence was confirmed morphologically by the formation of small clumps. These cells were then imaged in round glass-bottom dishes (35 mm dish with a 10 mm glass bottom, MatTek corporation) using an inverted laser-scanning confocal microscope (Zeiss 780 or 710) with a 63x/1.4 NA oil-immersion objective. The images were collected using Zen2010 software (Zeiss) and processed using Fiji or Image J. A modified version of under-agarose assay was used, as described previously (3–5). A thin layer of SeaKem GTG agarose (Lonza Biochemicals) in KK2MC (different concentrations to prepare gels of

different stiffnesses chiefly 0.5%, 0.75%, 1% and 2% w/v) of height 2 mm was poured in a preheated round glass-bottom dish (MatTek). Two parallel rectangular troughs of sizes 4 mm by 8 mm and 1 mm by 5 mm were cut in the gel, once it solidified. A gradient of cyclic-AMP was formed in the chamber by adding 5 µM cyclic-AMP to the larger well and leaving it for about 30 minutes. Once the gradient formed, $2x10^5$ cells/ml were added to the smaller trough and allowed to migrate. Load was applied using a cell squasher, as described previously (5), once the cells had migrated under the gel (about 40 minutes). Briefly, the plunger was bought in close contact with the surface of the gel by manually positioning it using micrometers and subsequently, a command to apply the load was given. A log file recorded the applied load and the position of the plunger.

Chemotaxis assay

Chemotactic ability of aggregation-competent cells was analysed by under-agarose assay, as described above (3, 4). The migration of aggregation-competent cells under an overlay of agarose (0.5% or 2% w/v) was imaged by low-magnification 20X objective on Zeiss 710 confocal microscope. The images were collected every 20 seconds for 20 minutes.

Blebbing assay

This assay was adapted as previously described (6). Aggregation competent cells at $2x10^5$/ml in 200 µl KK2MC were allowed to attach to a glass-bottom dish for 20 minutes, after which they were imaged by confocal microscope. 16 µl of 50 µm cyclic-AMP was added to the buffer while recording cells at 2fps.

Image analysis

Blebs and pseudopodia were identified by their morphological characteristics. Additionally, they were confirmed using kymographs where blebs could be seen as fast projections, devoid of actin polymerization at the leading edge whereas pseudopodia were identified by their characteristic slow actin dynamics (Figure 1B). In the movies where load was applied dynamically, blebs were scored at their first occurrence and the total number of blebs was binned in 1 second or 1 minute intervals.

Speed of cells was calculated by automated tracking using QUIMP plug-in in Image J (7). The centroid of cells was tracked at each frame of the movie to calculate the total distance travelled by the cell and divided by total time to calculate their speed.

Cell height was measured by reconstruction of the z-stacks (taken at 0.4 μm increments) using Imaris (Bitplane). Z-axis elongation which occurs due to mismatch in the refractive index (8) was corrected by comparing the true and apparent height of a fluorescent bead of known height (9.7 µm diameter Fluospheres; Molecular probes). The stacks were corrected by dividing them by 1.97 (giving a true z-axis increment of 0.203 μm). Statistical analysis of cell height was done using One Way ANOVA and Tukey's means comparison test in Origin software (Originlabs). Cell volume was calculated from the reconstructed stacks by calculating the volume of individual voxels occupied by the cells. Surface area was measured by calculating the total area of voxels on the outer surface. Fluorescence intensity was measured as the total florescence intensity of all relevant voxels.

Cell volume by FXM method was measured as described previously in (9). Aggregation competent *Dictyostelium* cells were confined using PDMS micropillars of heights 6 μm (corresponding to no load condition), 4 μm and 3 μm (corresponding to

the cell height under load or 2% agarose). The media around the cells contained 1mg/ml FITC-dextran which was displaced by the cells and the drop in fluorescence intensity was quantified using a custom MATLAB program, to measure the volume of the cells.

Cells were tracked using Manual Tracking plug-in in Image J with a centre correction option. The tracks were further analysed for chemotaxis by the Chemotaxis plug-in for ImageJ (NIH). The tracks were re-plotted with their origin as starting point. Euclidian distance was calculated as the difference between the starting and end points of the cell track, while accumulated distance followed the whole track. Speed was calculated by dividing accumulated distance by total time. Persistence is defined as the ratio between Euclidian distance and accumulated distance and chemotactic index as the cosine of the angle between net distance travelled in the direction of the gradient and the Euclidean distance.

The distribution and localization of myosin was analysed using a MATLAB plug-in kindly provided by Douwe Veltman and described in (10). A typical example of the analysis is shown in Supplementary figure S3. The plug-in tracks the membrane of the cell by identifying points of maximum intensity at the cell edge. It then draws equidistant normals of 3 pixels in length at intervals along the cell boundary, and calculates the points of maximum intensity along these and their coordinates. The mean fluorescence intensity of the background is then subtracted from the membrane and cytosol respectively. Each maxima on the membrane is mapped onto a corresponding local cytosolic point to compute the ratio between membrane and

cytoplasm to give normalized fluorescence intensity values (Supplementary figure S3). The distribution of the fluorescent signal is also quantified by measuring its periodicity. In the cells shown in Supplementary figure S3, the top cell (Supplementary figure S3A) has signal localized in restricted regions, while in the bottom cell (Supplementary figure S3B), the signal is localized homogeneously throughout the cell. The distribution can be described by a periodic function, and the Fourier transform of the signal defined as:

$$Y = \sum_{n=0}^{\infty} a_n \cos(2\pi nS/L) + \sum_{n=0}^{\infty} b_n \sin(2\pi nS/L)$$

with the ratio of two terms calculated as:

$$\sqrt{(a_1{}^2 + b_1{}^2)}\,/a_0$$

where S: position around cell contour

L: perimeter

The first term of the series is mean value of the function while the second one is the trigonometric function. The ratio of the first two terms of the series evaluates the difference in the peaks of the described signal. The more heterogeneous the signal, the larger is the gap between the peaks and as a result, larger is the ratio. In polarized cells the signal is strongly localized to only part of the cell and hence the intensity peaks are further apart, thereby giving a higher ratio between the terms. If myosin is more homogeneously localized in the cell, then the peaks are clustered together and a lower value of the ratio is expected. The robustness of this method was checked using the cells shown in Supplementary figure S3 and indeed the ratio of the first two terms for a polarized cell (Supplementary figure S3A) was 0.22 while that for un-polarized cell (Supplementary figure S3B) was 0.03.

The lifetime of an actin scar left behind by a bleb was measured from a kymograph taken along the line indicated in Supplementary figure S2A; the tracks demarcating the newly formed bleb (i_{bleb}), membrane (i_{memb}) and cytoplasm (i_{cyto}) are indicated (Supplementary figure S2A). A python script was written to calculate the intensity along the indicated tracks. In order to minimize the error due to fluctuation of fluorescence intensity with position, average intensity values along a line width of 3 pixels was taken. The intensity of scar is then defined as:

$$i_{scar} = i_{memb} - (i_{bleb} + i_{cyto}) / 2$$

The intensity of the scar is fitted with a sigmoid curve to obtain its half-life. The sigmoid curve used for fitting is:

$$Y = \frac{a}{1 + e^{-k(x+x_0)}}$$

and critical time obtained from the fit is given by

$$x_{crit} = x_0 - \frac{\log(1 + 2e^{kx_0})}{k}$$

Supplementary figure legends

Supplementary figure 1: The switch to bleb-driven motility caused by uniaxial load is reversible.

(A) Recovery of cells upon removal of external load. Cells revert from blebbing to forming pseudopodia when load is removed. A load of 400 Pa was applied to cells underneath 0.5% agarose gels and removed at t = 0.; Scale bar = 10 µm. (B) Quantification of blebs produced by cells under load and after its removal (after ~10 min). Data is mean ± SD for n≥15 cells tracked before and after the load in each case, p<0.0005 and one-way ANOVA. (C) Montage of cells after removal of load**.** Wild-type Ax2 cells were compressed by uniaxial 400 Pa and consequently switch to blebbing. Upon removal of load, these cells switch back to forming pseudopodia and adopt a polarised shape after about 10 min; scale bar = 10 µm.

Supplementary figure 2: Analysis of the actin scar left behind when a bleb is projected.

(A) A cell blebbing under a load of 400 Pa is shown with three distinct regions around a bleb marked as i_{bleb}, i_{memb} and i_{cyto} representing the newly formed bleb, the scar left behind by the bleb and cytoplasm of the cell, respectively; Scale bar = 10 µm. (B) Kymograph derived from the line indicated through the bleb shows its progression. Average fluorescence intensity for membrane, cytoplasm and bleb are measured along the paths indicated. (C) Plots of the average fluorescence intensities for

membrane, cytoplasm and bleb. Average fluorescence intensity calculated for the actin scar is fitted with a sigmoid function to obtain the half-life.

Supplementary figure 3: Analysis of the distribution of myosin-II using Fourier analysis.

A representative (A) polarized cell and an (B) less polarized cell. The graph indicates the variation in the fluorescence intensity of GFP-MhcA signal at the cell periphery, with a dashed line indicating the average intensity of the measured signal. After Fourier analysis of these intensity values, the ratio of the first and the second term defines the distribution of the intensity signal and thus, the polarity of the cell (see Materials and Methods).

Supplementary figure 4: Uniaxial loading causes changes in myosin-II distribution.

(A) Time course of changes in cortical GFP-MhcA polarity under load. Data is mean ± SEM for 10 cells, each tracked as load is applied starting at 0 sec; one-way ANOVA, $p<0.005$. Fourier transform of membrane fluorescence intensity values is used to quantify the polarity of cells (see supplementary Figure 4 for details). (B) Quantification of GFP-MhcA cortical enrichment under load. Data is represented as mean ± SD for $n \geq 20$ cells analysed for each case, one-way ANOVA, $p<0.005$. (C) Dynamics of myosin-II enrichment in cells expressing GFP-MhcA migrating under stiff 2% agarose gel; Scale bar = 10 µm. (D) Myosin-II null cells fail to bleb under uniaxial load, arguing that blebbing is an active process requiring contractility generated by myosin-II. Uniaxial load of 400 Pa was applied to MhcA null cells at t=0. The cells are

expressing the F-actin reporter, ABD-120 GFP, and are under 0.5% agarose gel; scale bar: 10 µm.

Supplementary figure 5: Effect of cyclic-AMP and ionomycin on cytoplasmic calcium levels and the blebbing response of wild-type and Piezo null cells.

(A) Cytoplasmic calcium levels are elevated following the addition of cyclic-AMP or ionomycin to wild-type Ax2 cells. Cytosolic calcium levels were measured using the Cameleon FRET-based sensor, with the normalised ratio of YFP 535nm/CFP485nm indicating the cytosolic calcium concentration (n = 20 cells). (B) Cyclic-AMP addition causes transient blebbing in a both wild-type cells expressing LifeAct-RFP and PzoA$^-$ cells expressing LifeAct-GFP, scale bar: 10 µm.

Supplementary figure 6: Effect of uniaxial loading on mutant cells lacking potential stretch-sensitive ion channels.

(A) Triple mutant of MscS, mucolipin (MclN) and TrpP channel. (B) Double mutant of IplA and TrpP channel. (C) Quantification of blebbing after application of uniaxial load to strains mutant for candidate mechanosensitive ion channels and wild-type Ax2 cells. Scale bars are 10 µm.

Supplementary figure 7: Creation of PzoA knock-out strains.

(A) PzoA knock-out strains were created using the plasmid pDT43 (plasmid design by Macvector software). (B) A diagnostic PCR showing two positive clones, which yield a band at around 4.5 kB, which is sensitive to digestion with SmaI. This liberates the 5’

and 3’ homology arms together with the blasticidin cassette, while in wild-type cells no product was obtained.

Supplementary figure 8: Piezo is required for sensing compressive load.

(A) Piezo null cells (PzoA$^-$) do not respond to uniaxial load by blebbing. Load is applied to a mixture of PzoA$^-$ cells expressing LifeAct-GFP and wild-type cells expressing LifeAct-mRFP under 0.5% agarose. PzoA$^-$ cells continue to migrate with pseudopods when the load is applied but wild-type cells form blebs instead (indicated by *) (Scale bar:10 µm). (B) Greater uniaxial loading does not cause blebbing in PzoA$^-$ cells. PzoA$^-$ cells were subjected to 800 Pa and 1600 Pa load but continued to migrate with pseudopods; Scale bar = 10 µm. (C) Increasing the stiffness of agarose gels does not cause a switch to blebbing in PzoA$^-$ cells. A mixture of PzoA$^-$ cells expressing LifeAct-GFP and wild-type cells expressing LifeAct-mRFP were induced to chemotax under stiffer 1% and 2% agarose gels. PzoA$^-$ cells moved with fewer blebs compared to wild-type cells; Scale bar = 10 µm. Quantification of blebs formed by wild-type and PzoA$^-$ cells under (D) stiff agarose gels and (E) compressive load. The data is represented as mean ± SD for n ≥ 30 cells for each case.

Supplementary figure 9: HM1813, the second Piezo mutant clone, does not bleb under load. (A) Piezo null clone (HM1813) does not respond to uniaxial load. Load is applied to PzoA$^-$ cells expressing LifeAct-GFP under 400 Pa load. PzoA$^-$ cells continue to form pseudopods under load; Scale bar = 10 µm. (B) Quantification of the blebs formed by PzoA$^-$ cells under compressive load. The data is represented as mean ± SD for n ≥ 20 cells for each case.

Supplementary figure 10: Myosin distribution does not change under compressive load in PzoA$^-$ cells. (A) Myosin dynamics in PzoA$^-$ expressing RFP-myosin-II (RFP-MhcA) during loading. Load was applied to cells chemotaxing under 0.5% agarose gel. Time is given with respect to the start of loading (t=0); blebs are indicated by * and scale bar =10 µm. (B) Time course of RFP-MhcA recruitment to the cell cortex under load in PzoA$^-$ cells. (C) RFP-MhcA distribution under load in PzoA$^-$ cells. Time is given with respect to the start of loading (t=0). Data is represented as mean ± SEM, n=10 cells, one-way ANOVA, $p>0.5$ in both cases. (D) Quantification of the cortical distribution of myosin-II in wild-type and Piezo null cells under a load of 400 Pa. The data is represented as mean ± SD for n≥20 cells analysed for each case, $p<0.005$ for wild-type cells and $p>0.5$ for Piezo null cells, Mann-Whitney test and one-way ANOVA.

**Cell Parameter**	**Control (0.5% agarose)**	**400 Pa load (0.5% agarose)**	**Under 2% agarose (No load)**
Height	6.7 ± 1.2 μm	3.4 ± 0.7 μm	2.94 ± 0.72 μm
Volume (measured by confocal)	478.9 ± 70.1 μm^3	360.9 ± 58.3 μm^3	
Mean fluorescence intensity of cytosolic GFP	35.7 ± 8.3 AU	65.5 ± 28.6 AU	
Surface area	472.2 ± 55.1 μm^2	343.7 ± 52.6 μm^2	
Actin scar half-life	4.3 ± 0.6 sec	10.6 ± 0.9 sec	
Cortical myosin	1.63 ± 0.18 AU	2.8 ± 0.96 AU	2.88 ± 0.21 AU
Cortical myosin Piezo null mutant	1.50 ± 0.1 AU	1.3 ± 0.1 AU	
Polarity	0.17 ± 0.04 AU	0.1 ± 0.05 AU	0.27 ± 0.19 AU
Volume (measured by fluorescence exclusion method)	560 ± 166 μm^3(6 μm confinement)	420 ± 130 μm^3(4 μm confinement)	420 ± 120μm^3(3 μm confinement)

Table1: The effect of load, overlay stiffness and confinement on cellular parameters.

The main body of the table shows measurements made in the standard conditions under an agarose overlay. The bottom panel shows cells confined to different heights in a microfluidics chamber, with cell volume measured by a dye-exclusion method.

Main body: The effect of load or overlay stiffness on cellular parameters was determined in the conditions shown. Aggregation-competent cells migrating towards cyclic-AMP were used in all cases with either 0.5% or 2% agarose overlays, giving stiffness of 6.6 kPa and 73.6 Pa respectively. Ax2 cells were used, except where indicated (n>30 in all cases). The methods used to quantify cellular parameters are described in the Materials and Methods.

Bottom panel: The loss of volume upon imposition of load was further confirmed using a fluorescent dye exclusion method (9, 11). Aggregation-competent cells were prepared by starving them for about 6 hours in KK2 buffer. 100 μl of 10^6 cells/ml in KK2 buffer containing 1mg/ml FITC-dextran was confined in microfluic chambers of heights 6μm, 4μm and 3μm. The height of microfluidic chamber was fixed by PDMS micropillars. Ax2 cells were used in all the experiments (n>200 cells in 3 different experiments).

Caption for movies

S1: Cell moving under 0.5% agarose gel. Aggregation competent Ax2 cell, expressing ABD-GFP as a reporter for F-actin, chemotaxing to cyclic-AMP under 0.5% agarose without external mechanical load applied. Cells move predominantly with pseudopods and rarely with blebs under these conditions. Taken by a laser scanning confocal microscope (Zeiss 780) at 2 frames per second.

S2: Cell moving under 100 Pa compressive load and 0.5% agarose gel. An aggregation competent Ax2 cell expressing ABD-GFP as a reporter for F-actin, is chemotaxing to cyclic-AMP under 0.5% agarose and a compressive load of 100 Pa. The cell migrates with a combination of blebs (indicated by *) and pseudopods. Majority of blebs are formed at the leading edge. Taken by a laser scanning confocal microscope (Zeiss 780) at 2 frames per second about 8-10 minutes after the load is applied.

S3: Cell moving under 800 Pa compressive load and 0.5% agarose gel. An aggregation competent Ax2 cell expressing ABD-GFP as a reporter for F-actin, is chemotaxing to cyclic-AMP under 0.5% agarose and a compressive load of 400 Pa. The cell typically migrates with blebs (indicated by *) and infrequently with pseudopods. Taken by a laser scanning confocal microscope (Zeiss 780) at 2 frames per second about 8-10 minutes after the load is applied.

S4: Cell migrating under 1600 Pa compressive load and 0.5% agarose gel. An aggregation competent Ax2 cell expressing ABD-GFP as a reporter for F-actin, is chemotaxing to cyclic-AMP under 0.5% agarose and a compressive load of 1600 Pa. The cell typically projects numerous blebs (indicated by *) with a drastic decrease in the migratory speed. The blebs typically form all around the cells with a noticeable change in the cell shape. Taken by a laser scanning confocal microscope (Zeiss 780) at 2 frames per second about 8-10 minutes after the load is applied.

S5: Instantaneous response of a cell to a load of 400 Pa. An aggregation competent Ax2 cell expressing ABD-GFP as a reporter for F-actin is chemotaxing to cyclic-AMP under 0.5% agarose, at first without an external load. It is then suddenly subjected to a compressive load of 400 Pa. The cell initially moves by forming pseudopods under

0.5% agarose but starts to bleb (indicated by *) as soon as the load is applied. Taken by a laser scanning confocal microscope (Zeiss 780) at 2 frames per second.

S6: Switch to blebbing mode of migration is reversible upon removal of the compressive load. Aggregation competent Ax2 cells expressing LifeAct-mcherry as a reporter for F-actin and chemotaxing to cyclic-AMP under 0.5% agarose gel and 400 Pa compressive load show blebbing phenotype. Subsequently, the blebbing decreases as the load is removed. The cells revert back to pseudopod-based migration with a distinct polarized shape by about 10 min after removal of load. This clearly shows that the switch to blebbing is reversible. Taken by a laser scanning confocal microscope (Zeiss 780) at 2 frames per second in the beginning of the movie and 1 frame per 2 minutes after the removal of the load.

S7: Rapid recruitment of myosin to the cell cortex upon application of compressive load on cells. Aggregation competent Ax2 cells expressing GFP-MhcA as a reporter for myosin-II chemotaxing towards cyclic-AMP under 0.5% agarose gel. Myosin, which is localized mostly at the rear and sides of the migrating cell, rapidly relocalizes to the cortex in response to 400 Pa compressive load. The temporal dynamics of myosin localization is similar to that of protrusion switching from pseudopodia to blebs due to compressive loading. Taken by a laser scanning confocal microscope (Zeiss 780) at 2 frames per second.

S8: Cells do not switch to bleb driven migration under compressive loading in the absence of extracellular calcium.

Aggregation competent Ax2 cells expressing ABD-GFP as a reporter for F-actin, in calcium-free media to which 200 μM EGTA was added, chemotaxing towards cyclic-AMP under 0.5% agarose gel. The cells predominantly migrate with pseudopods

underneath the soft gel and do not switch to bleb-driven migration when a compressive load of 400 Pa is imposed. These cells continue to form pseudopods as they migrate. Taken by a laser scanning confocal microscope (Zeiss 780) at 2 frames per second.

S9: A triple mutant of mucolipin, TrpP and MscS channels migrating under a compressive load.

An aggregation competent triple mutant of the mechano-sensing channel MscS and two Trp channels (one a mucolipin homologue, MclN; the other, TrpP, responsive to ATP) expressing LifeAct-GFP as a reporter for F-actin, is chemotaxing to cyclic-AMP under 0.5% agarose and a compressive load of 400 Pa. The cell predominantly migrates by forming blebs and very infrequently with pseudopods. Taken by a laser scanning confocal microscope (Zeiss 780) at 2 frames per second about 8-10 minutes after the load is applied.

S10: Instantaneous response of a triple mutant of mucolipin, TrpP and MscS stretch-activated channels to a compressive load of 400 Pa.

Aggregation competent cells of the triple mutant of the mechano-sensing channel MscS and two Trp channels (one a mucolipin homologue, MclN; the other, TrpP, responsive to ATP) expressing LifeAct-GFP as a reporter for F-actin, is chemotaxing to cyclic-AMP under 0.5% agarose. The cell typically moves by forming pseudopods under the soft gel but rapidly switches to bleb-driven migration as a dynamic load of 400 Pa is imposed upon it. Taken by a laser scanning confocal microscope (Zeiss 780) at 2 frames per second.

S11: A double mutant of TrpP and IplA stretch-activated channels migrating under a compressive load.

An aggregation competent double mutant of the mechano-sensing channels: Trp channel (TrpP, responsive to ATP) and IplA (a homologue of the IP3 receptor required for the calcium response to chemoattractants), expressing LifeAct-GFP as a reporter for F-actin, is migrating under 0.5% agarose and a compressive load of 400 Pa, by predominantly forming blebs. Taken by a laser scanning confocal microscope (Zeiss 780) at 2 frames per second about 8-10 minutes after the load is applied.

S12: Instantaneous response of a double mutant of TrpP and IplA stretch-activated channels to a compressive load of 400 Pa.

An aggregation competent double mutant of the mechano-sensing channels: Trp channel (TrpP, responsive to ATP) and IplA (a homologue of the IP3 receptor required for the calcium response to chemoattractants), expressing LifeAct-GFP as a reporter for F-actin, is chemotaxing to cyclic-AMP under 0.5% agarose. The cell initially moves by forming pseudopods under the soft gel but switches to a bleb-driven migration as a load of 400 Pa is imposed upon it. The response to load by this mutant is similar to that of its WT parent. Taken by a laser scanning confocal microscope (Zeiss 780) at 2 frames per second.

S13: Instantaneous response of cells lacking the Piezo channel to a compressive load of 400 Pa. A mixture of aggregation-competent Ax2 cells expressing LifeAct-RFP and *pzoA*- null cells expressing LifeAct-GFP is chemotaxing towards cyclic-AMP underneath a 0.5% agarose overlay. A compressive load of 400 Pa applied to the cells causes a switch from pseudopodia to bleb-driven motility in Ax2 cells (blebs are indicated by *), whereas *pzoA*$^{-}$ mutants are unresponsive, and continue to form pseudopods. Taken by a laser scanning confocal microscope (Zeiss 780) at 2 frames per second.

S14: Migration of the other Piezo null clone (HM1813) cells under 0.5% agarose gel to which a 400 Pa compressive load is applied. The cells forms both pseudopods and blebs, unlike its wild-type parent, which exclusively forms blebs under the same conditions. Taken by a laser scanning confocal microscope (Zeiss 780) at 2 frames per second about 8-10 minutes after the load is applied.

S15: Migration of Piezo null cell under 0.5% agarose gel to which a 1600 Pa compressive load is applied. The cell forms both pseudopods and blebs, unlike its wild-type parent, which exclusively forms blebs under the same conditions. Taken by a laser scanning confocal microscope (Zeiss 780) at 2 frames per second about 8-10 minutes after the load is applied.

S16: Stimulation of blebbing by cyclic-AMP in wild-type and Piezo null cells. A mixture of aggregation competent Ax2 cells, expressing LifeAct-RFP, and Piezo null cells, expressing LifeAct-GFP is stimulated with a saturating dose of 4 μM cyclic-AMP. This causes both wild-type and Piezo mutants to transiently round up and copiously bleb, showing that Piezo null cells retain the ability to bleb in response to chemotactic stimulation but not mechanical load. Cell in in KK2 buffer imaged by laser scanning confocal microscopy (Zeiss 780) at 2 frames per second.

S17: The effect of compressive load on myosin-II distribution in Piezo null mutant cells. Aggregation competent Piezo null mutant cells expressing RFP-MhcA as a reporter for myosin-II chemotaxing towards cyclic-AMP under 0.5% agarose gel. Similar to the parent Ax2 cells, myosin-II in Piezo mutants localizes to the rear and side of the cells, but unlike the parent, the application of a load of 400 Pa does not

cause recruitment of myosin-II to the cortex. Taken by laser scanning confocal microscopy (Zeiss 780) at 2 frames per second.

References

1. K. M. Pang, E. Lee, D. a Knecht, Use of a fusion protein between GFP and an actin-binding domain to visualize transient filamentous-actin structures. *Curr. Biol.* **8**, 405–8 (1998).

2. D. M. Veltman, G. Akar, L. Bosgraaf, P. J. M. M. Van Haastert, A new set of small, extrachromosomal expression vectors for *Dictyostelium* discoideum. *Plasmid* **61**, 110–118 (2009).

3. G. Laevsky, D. a Knecht, Under-agarose folate chemotaxis of *Dictyostelium* discoideum amoebae in permissive and mechanically inhibited conditions. *Biotechniques* **31**, 1140–1142, 1144, 1146–1149 (2001).

4. E. Zatulovskiy, R. Tyson, T. Bretschneider, R. R. Kay, Bleb-driven chemotaxis of *Dictyostelium* cells. *J. Cell Biol.* **204**, 1027–1044 (2014).

5. N. Srivastava, R. R. Kay, A. J. Kabla, Method to study cell migration under uniaxial compression. *Mol. Biol. Cell* **28**, 809–816 (2017).

6. P. D. Langridge, R. R. Kay, Blebbing of *Dictyostelium* cells in response to chemoattractant. *Exp. Cell Res.* **312**, 2009–17 (2006).

7. R. A. Tyson, D. B. A. Epstein, K. I. Anderson, T. Bretschneider, High Resolution Tracking of Cell Membrane Dynamics in Moving Cells: an Electrifying Approach. *Math. Model. Nat. Phenom.* **5**, 34–55 (2010).

8. S. W. Hell, G. Reiner, C. Cremer, E. H. K. Stelzer, Aberrations in Confocal Fluorescence Microscopy Induced by Mistakes in Refractive Index. *J Microsc* **169**, 391–405 (1993).

9. E. Zlotek-Zlotkiewicz, S. Monnier, G. Cappello, M. Le Berre, M. Piel, Optical volume and mass measurements show that mammalian cells swell during

mitosis. *J. Cell Biol.* **211**, 765–74 (2015).

10. G. Bloomfield, *et al.*, Neurofibromin controls macropinocytosis and phagocytosis in *Dictyostelium*. *Elife* **4**, 4419–4427 (2015).

11. C. Cadart, *et al.*, “Fluorescence eXclusion Measurement of volume in live cells” in *Methods in Cell Biology*, (2017), pp. 103–120.

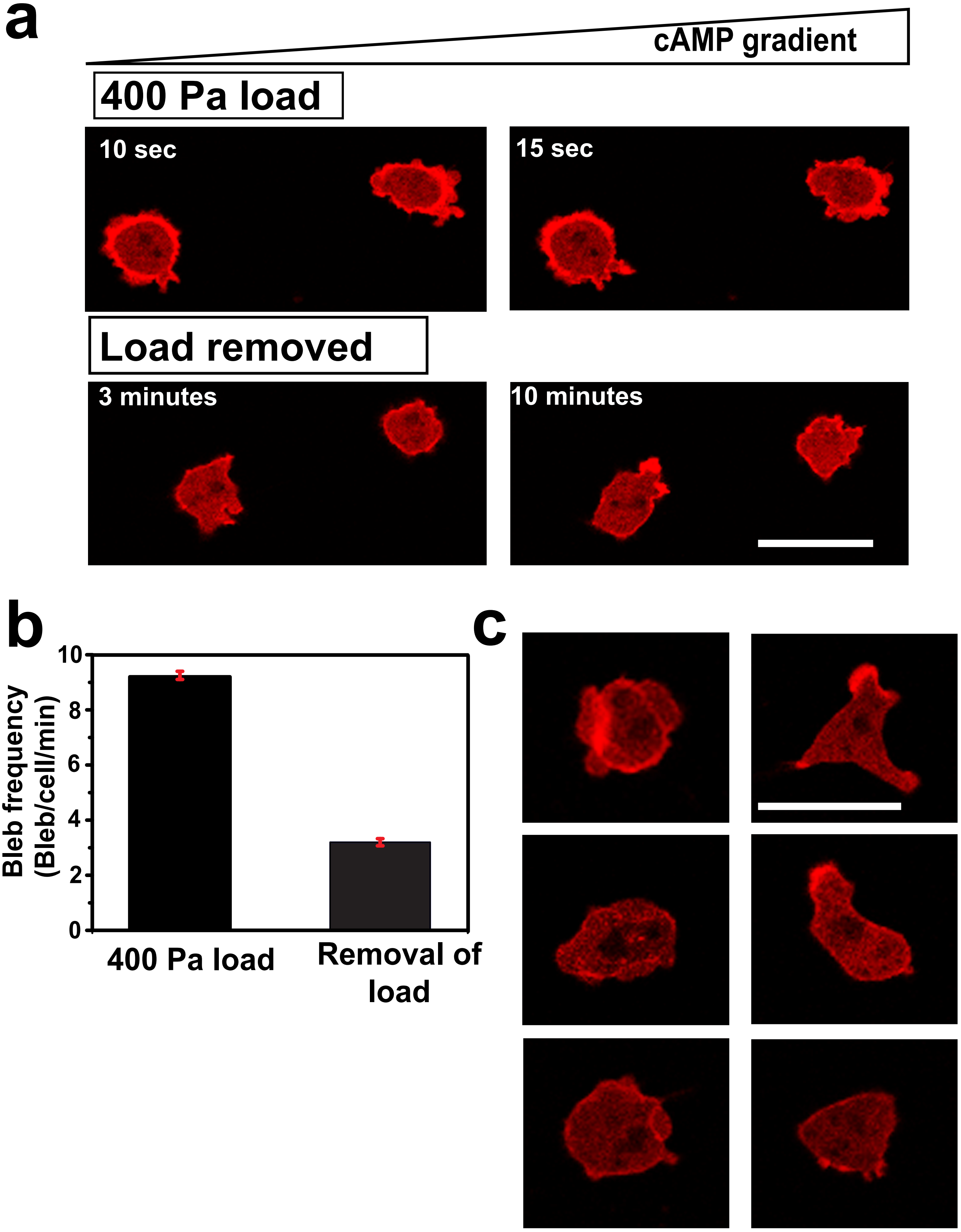

a
cAMP gradient
400 Pa load
10 sec
15 sec
Load removed
3 minutes
10 minutes
b
Bleb frequency (Bleb/cell/min)
0
2
4
6
8
10
400 Pa load
Removal of load
c

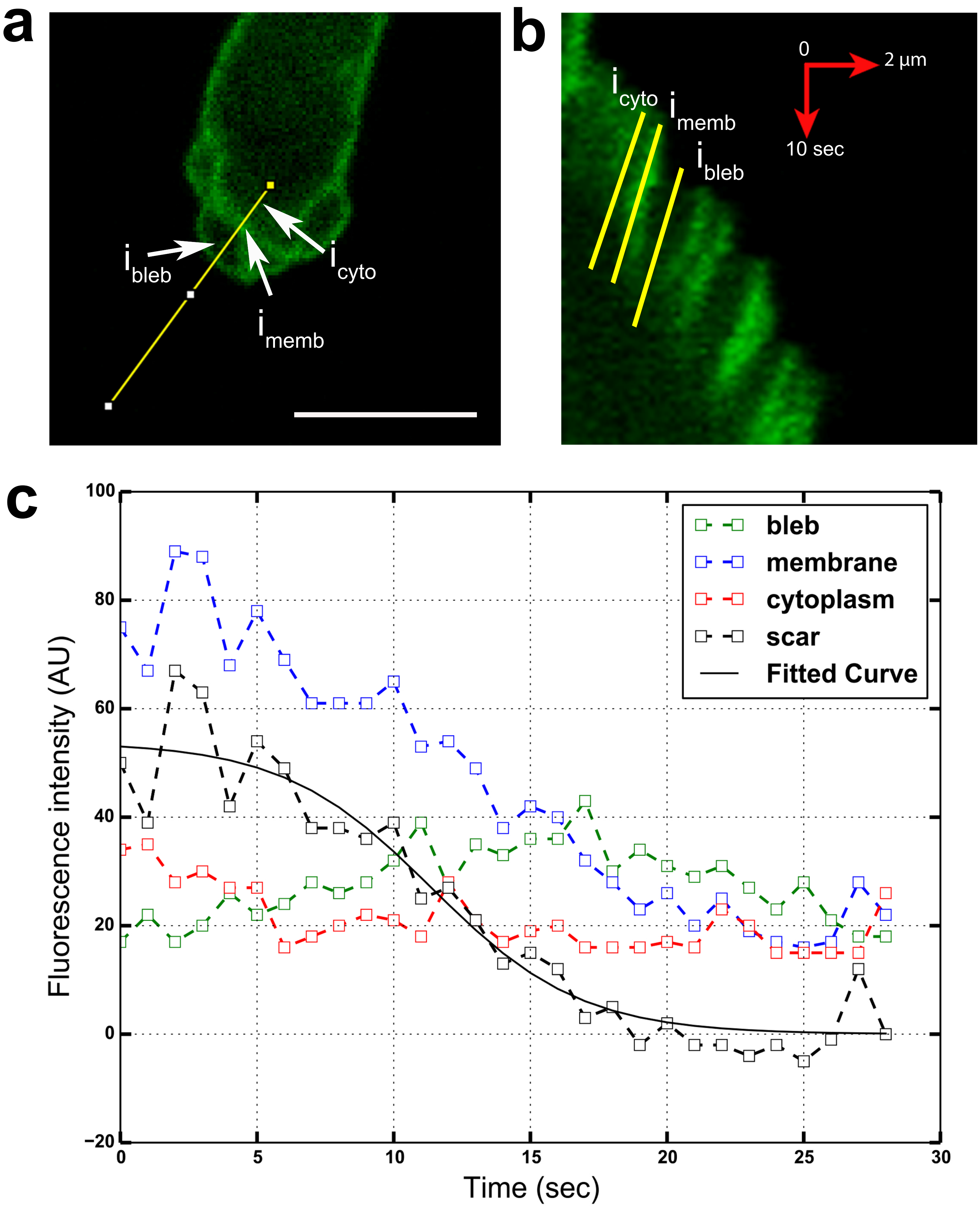

a
i bleb
i cyto
i memb
b
i cyto
i memb
i bleb
0
2 μm
10 sec
c
bleb
membrane
cytoplasm
scar
Fitted Curve
Fluorescence intensity (AU)
Time (sec)
100
80
60
40
20
0
−20
0
5
10
15
20
25
30

a

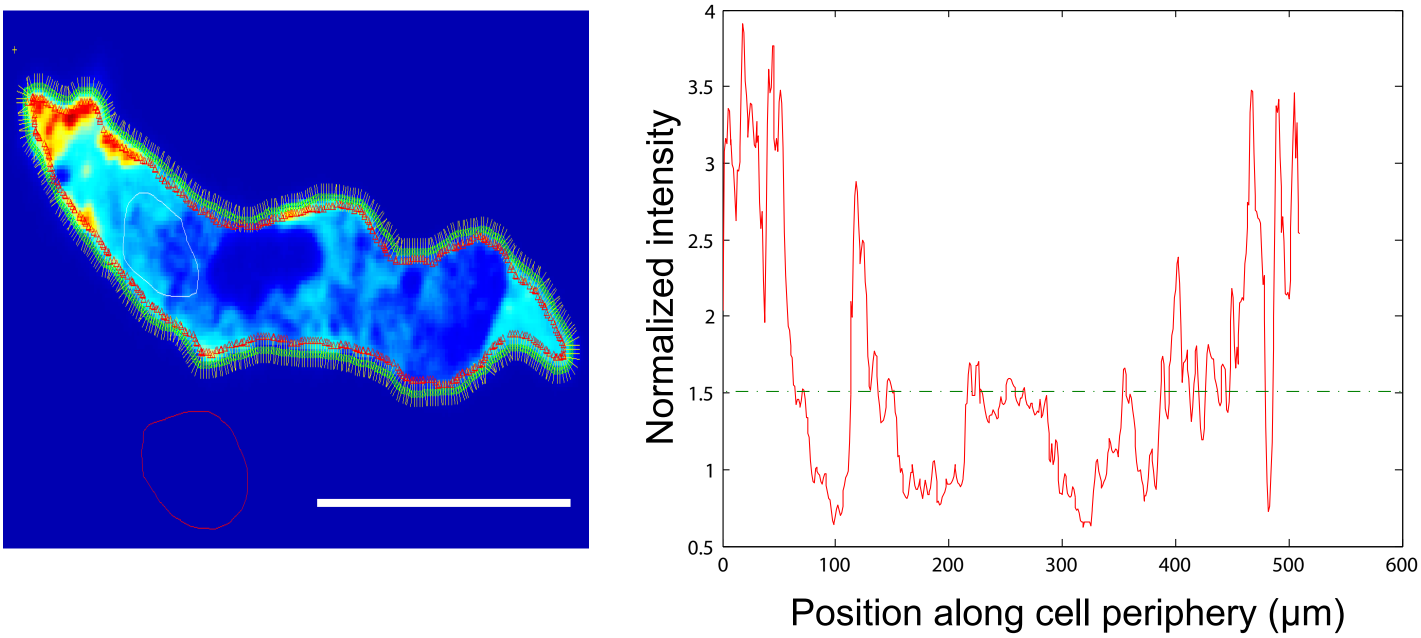
Normalized intensity
Position along cell periphery (μm)

b

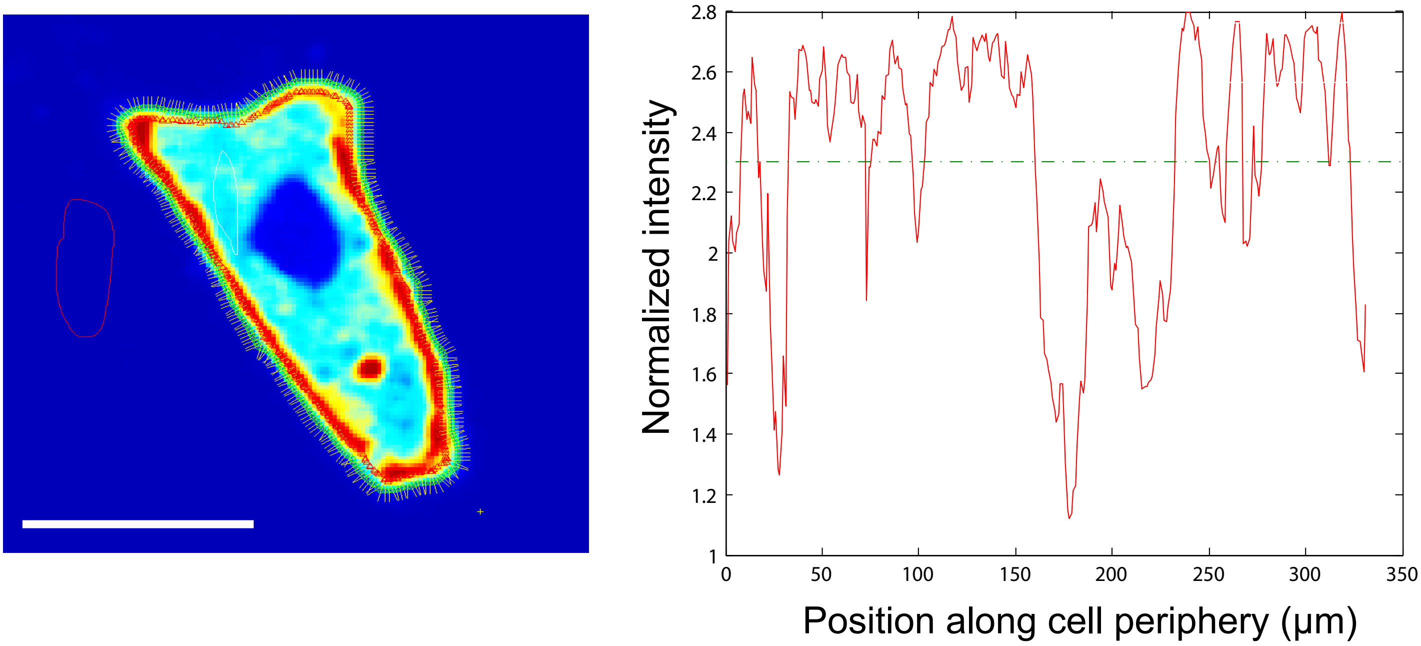
Normalized intensity
Position along cell periphery (μm)

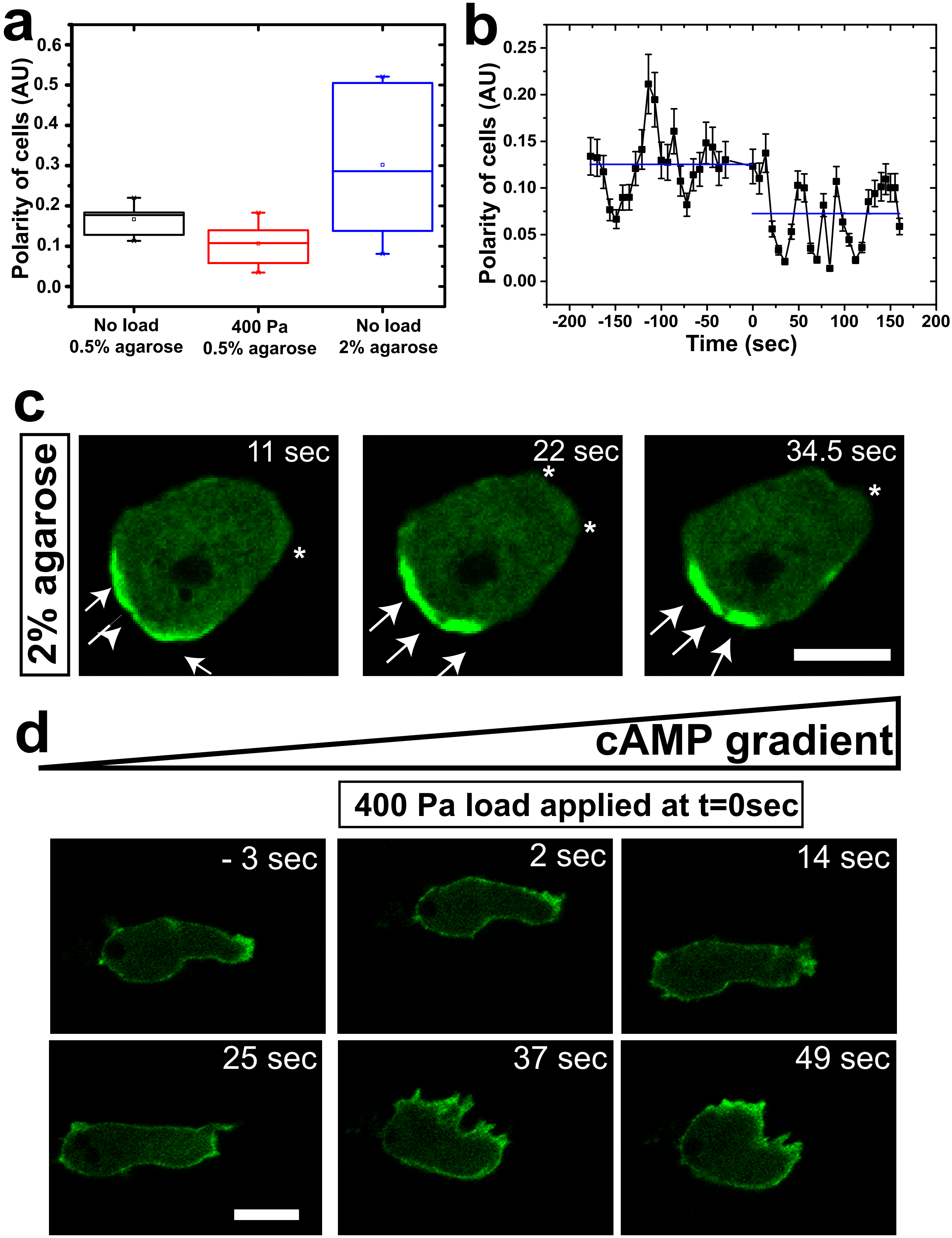
a
Polarity of cells (AU)
0.6
0.5
0.4
0.3
0.2
0.1
0.0
No load
0.5% agarose
400 Pa
0.5% agarose
No load
2% agarose
b
Polarity of cells (AU)
0.25
0.20
0.15
0.10
0.05
0.00
-200
-150
-100
-50
0
50
100
150
200
Time (sec)
c
2% agarose
11 sec
22 sec
34.5 sec
d
cAMP gradient
400 Pa load applied at t=0sec
- 3 sec
2 sec
14 sec
25 sec
37 sec
49 sec

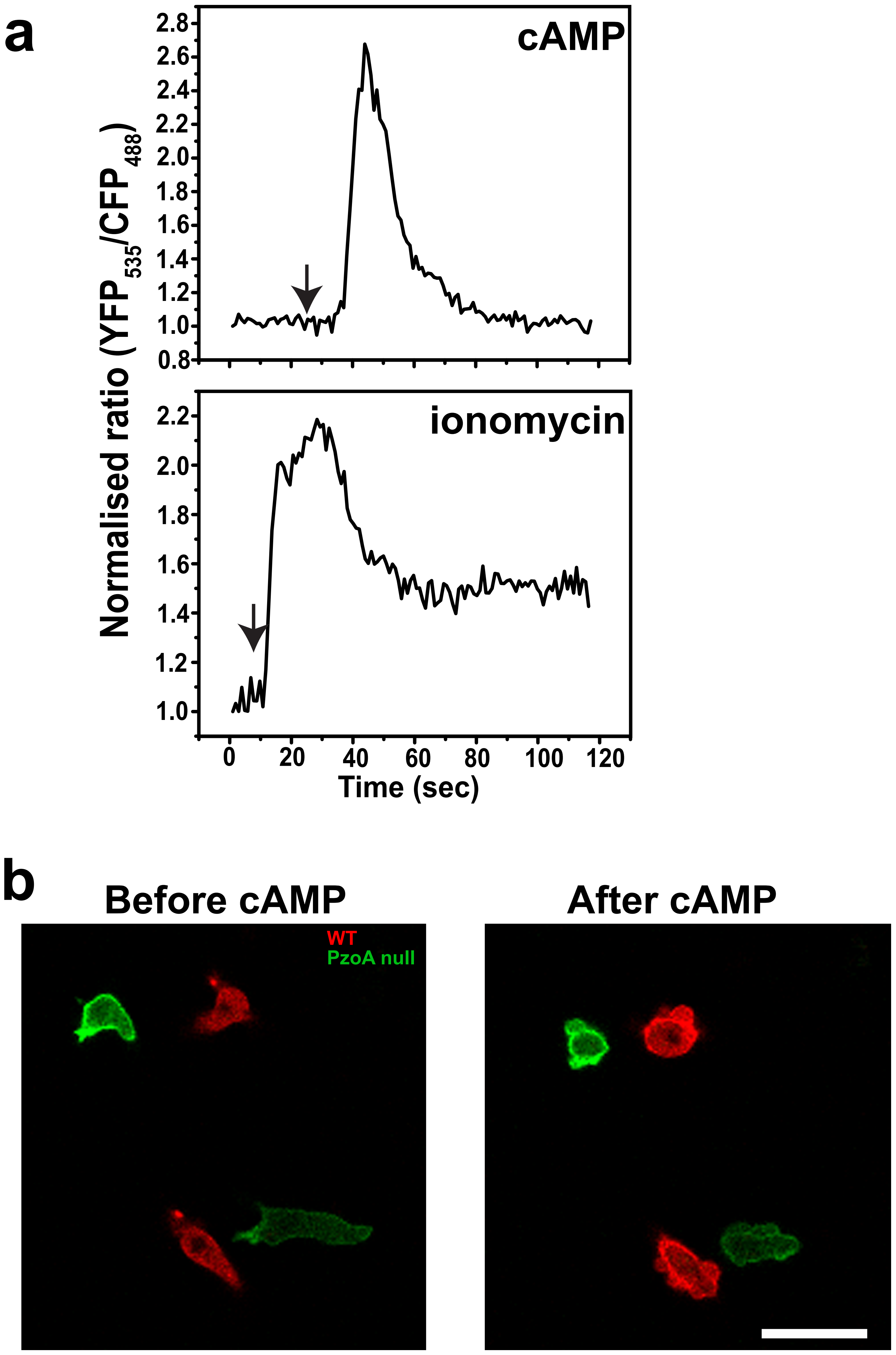
a
cAMP
ionomycin
Normalised ratio (YFP535/CFP488)
Time (sec)
0
20
40
60
80
100
120
0.8
1.0
1.2
1.4
1.6
1.8
2.0
2.2
2.4
2.6
2.8
b
Before cAMP
After cAMP
WT
PzoA null

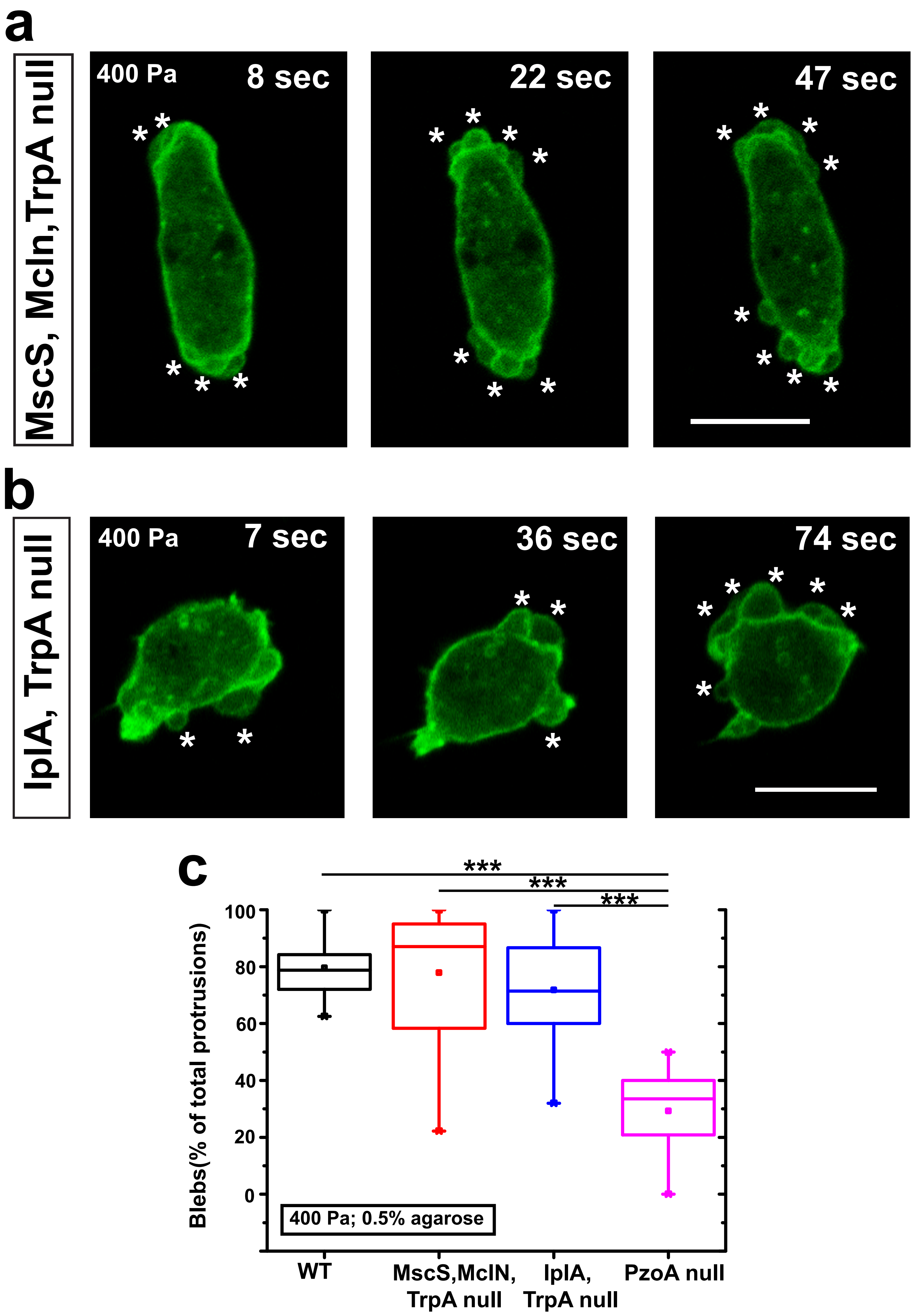

a
MscS, McIn,TrpA null
400 Pa
8 sec
22 sec
47 sec
b
IplA, TrpA null
400 Pa
7 sec
36 sec
74 sec
c
***
***
***
Blebs(% of total protrusions)
100
80
60
40
20
0
400 Pa; 0.5% agarose
WT
MscS,McIN, TrpA null
IplA, TrpA null
PzoA null

a

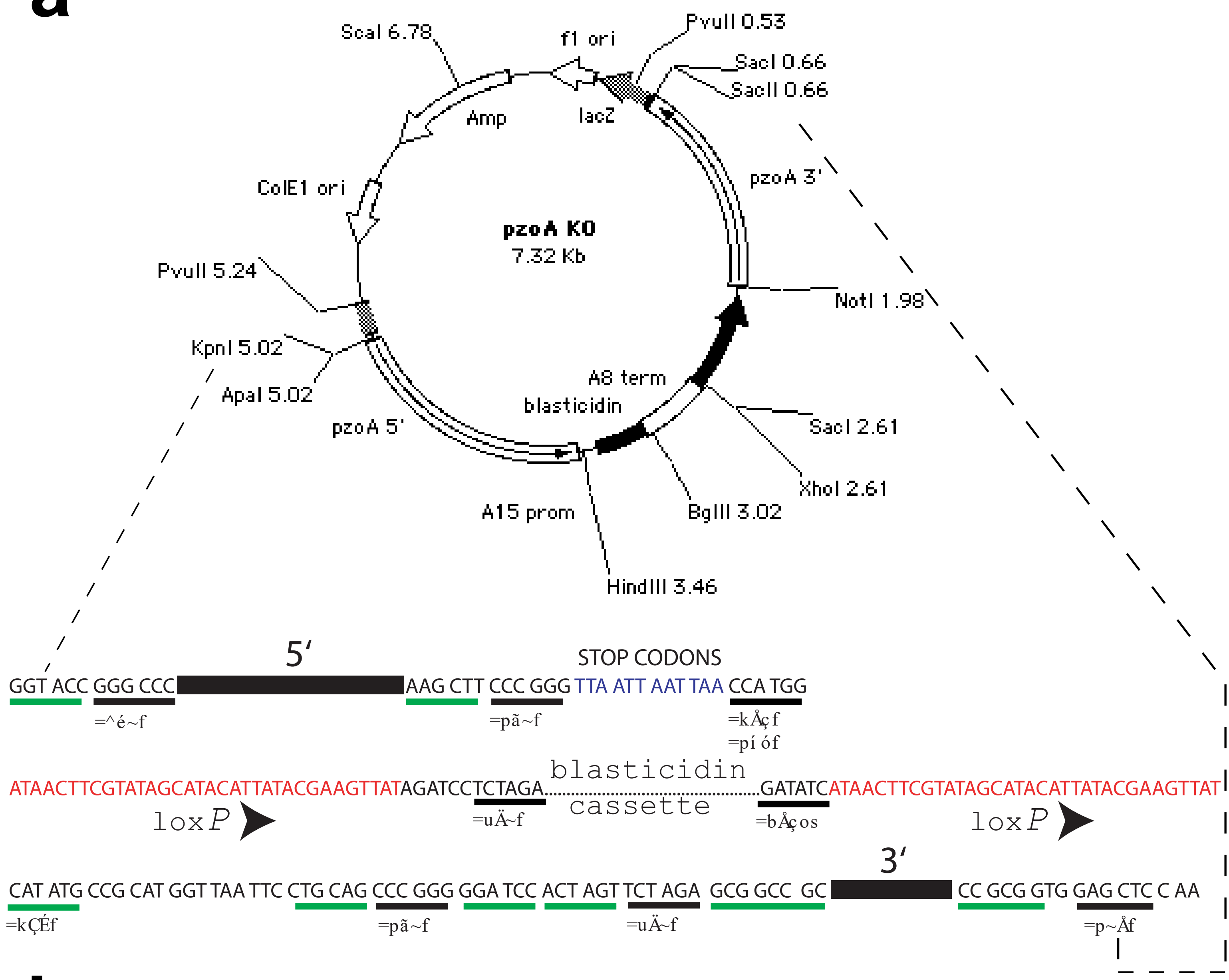
ScaI 6.78
f1 ori
PvuII 0.53
SacI 0.66
SacII 0.66
Amp
lacZ
ColE1 ori
pzoA 3'
pzoA KO
7.32 Kb
PvuII 5.24
NotI 1.98
KpnI 5.02
ApaI 5.02
A8 term
blasticidin
pzoA 5'
SacI 2.61
XhoI 2.61
A15 prom
BglII 3.02
HindIII 3.46
5'
STOP CODONS
GGT ACC GGG CCC AAG CTT CCC GGG TTA ATT AAT TAA CCA TGG
=^é~f
=pã~f
=kÅç f
=pí óf
ATAACTTCGTATAGCATACATTATACGAAGTTATAGATCCTCTAGA blasticidin cassette GATATCATAACTTCGTATAGCATACATTATACGAAGTTAT
lox P
=uÄ~f
=bÅç os
lox P
3'
CAT ATG CCG CAT GGT TAA TTC CTG CAG CCC GGG GGA TCC ACT AGT TCT AGA GCG GCC GC CC GCG GTG GAG CTC C AA
=kÇÉf
=pã~f
=uÄ~f
=p~Åf

b

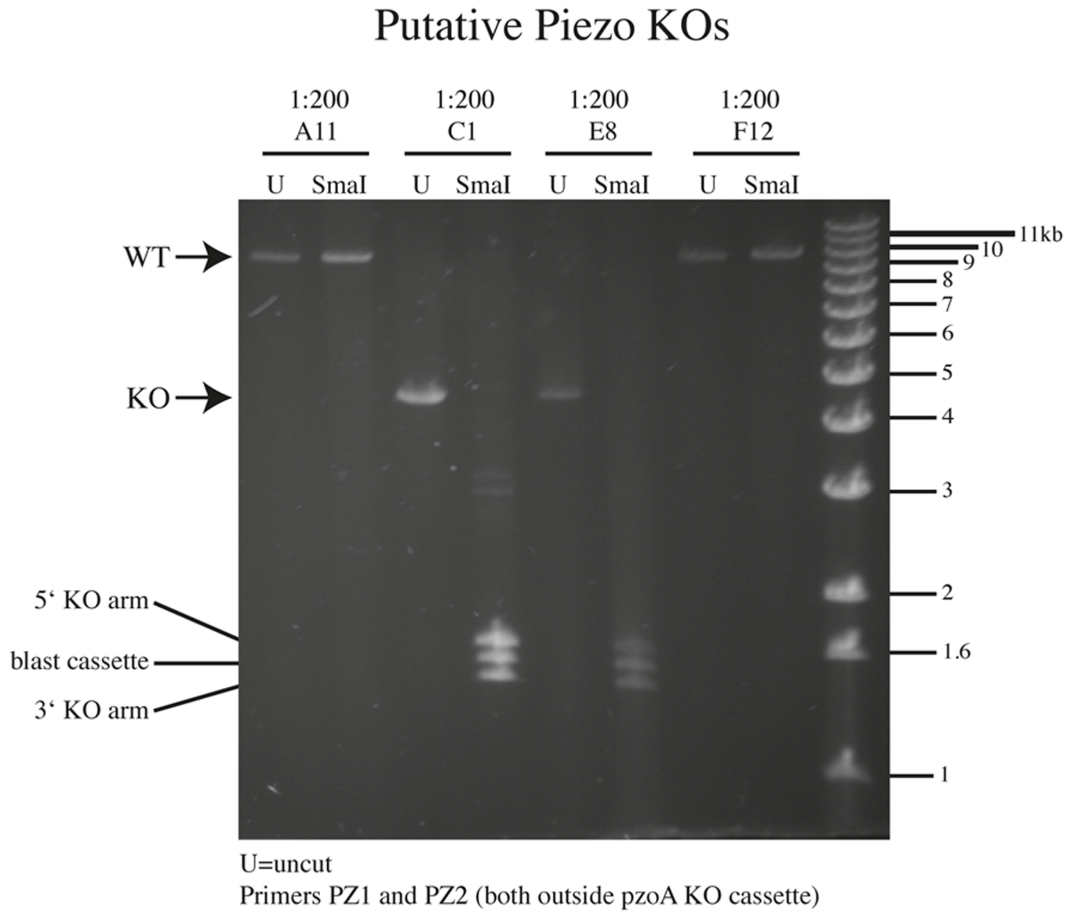
Putative Piezo KOs
1:200
A11
1:200
C1
1:200
E8
1:200
F12
U SmaI U SmaI U SmaI U SmaI
WT
KO
5' KO arm
blast cassette
3' KO arm
11kb
10
9
8
7
6
5
4
3
2
1.6
1
U=uncut
Primers PZ1 and PZ2 (both outside pzoA KO cassette)

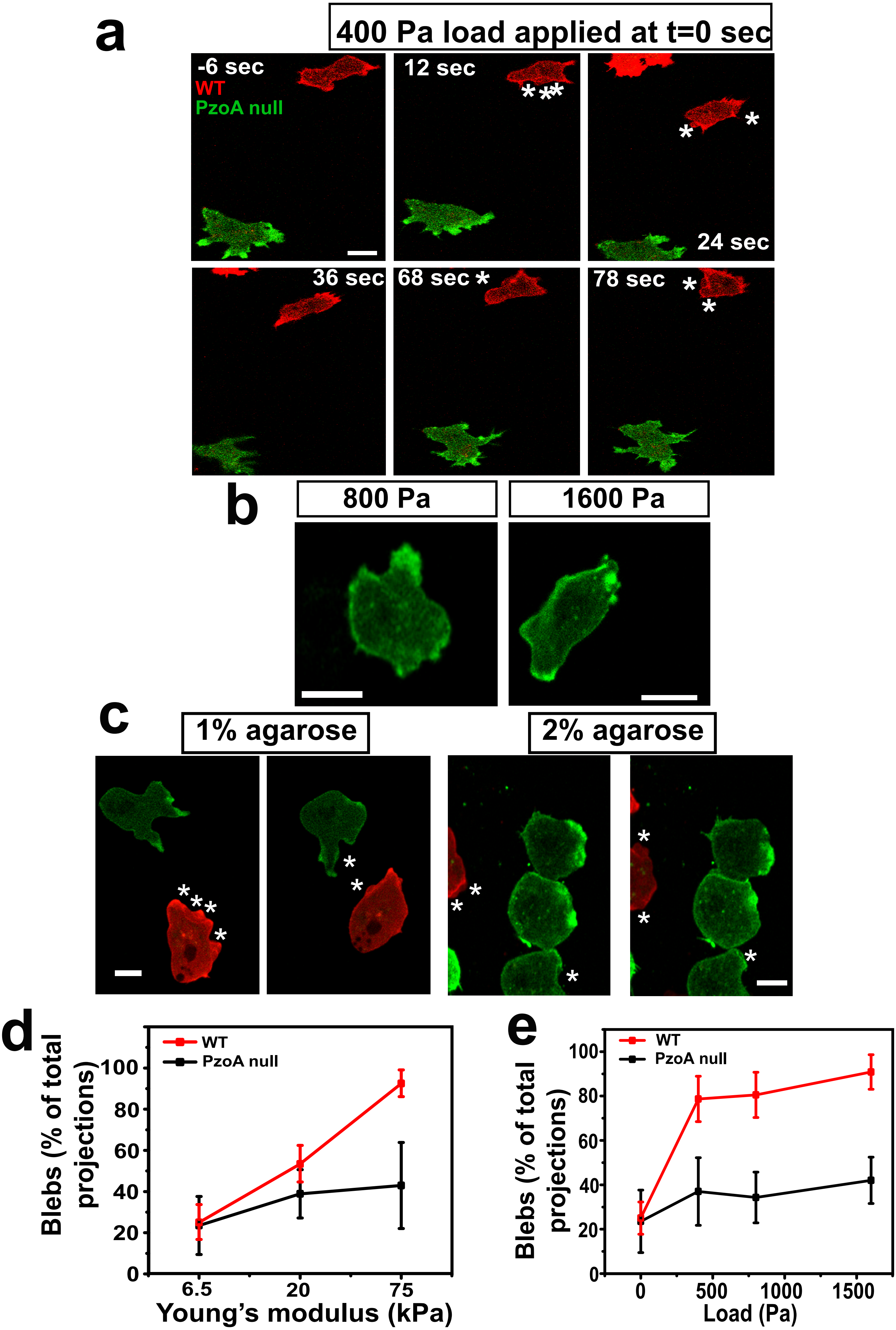
a
400 Pa load applied at t=0 sec
-6 sec
WT
PzoA null
12 sec
24 sec
36 sec
68 sec
78 sec
b
800 Pa
1600 Pa
c
1% agarose
2% agarose
d
WT
PzoA null
Blebs (% of total projections)
0
20
40
60
80
100
6.5
20
75
Young's modulus (kPa)
e
WT
PzoA null
Blebs (% of total projections)
0
20
40
60
80
100
0
500
1000
1500
Load (Pa)

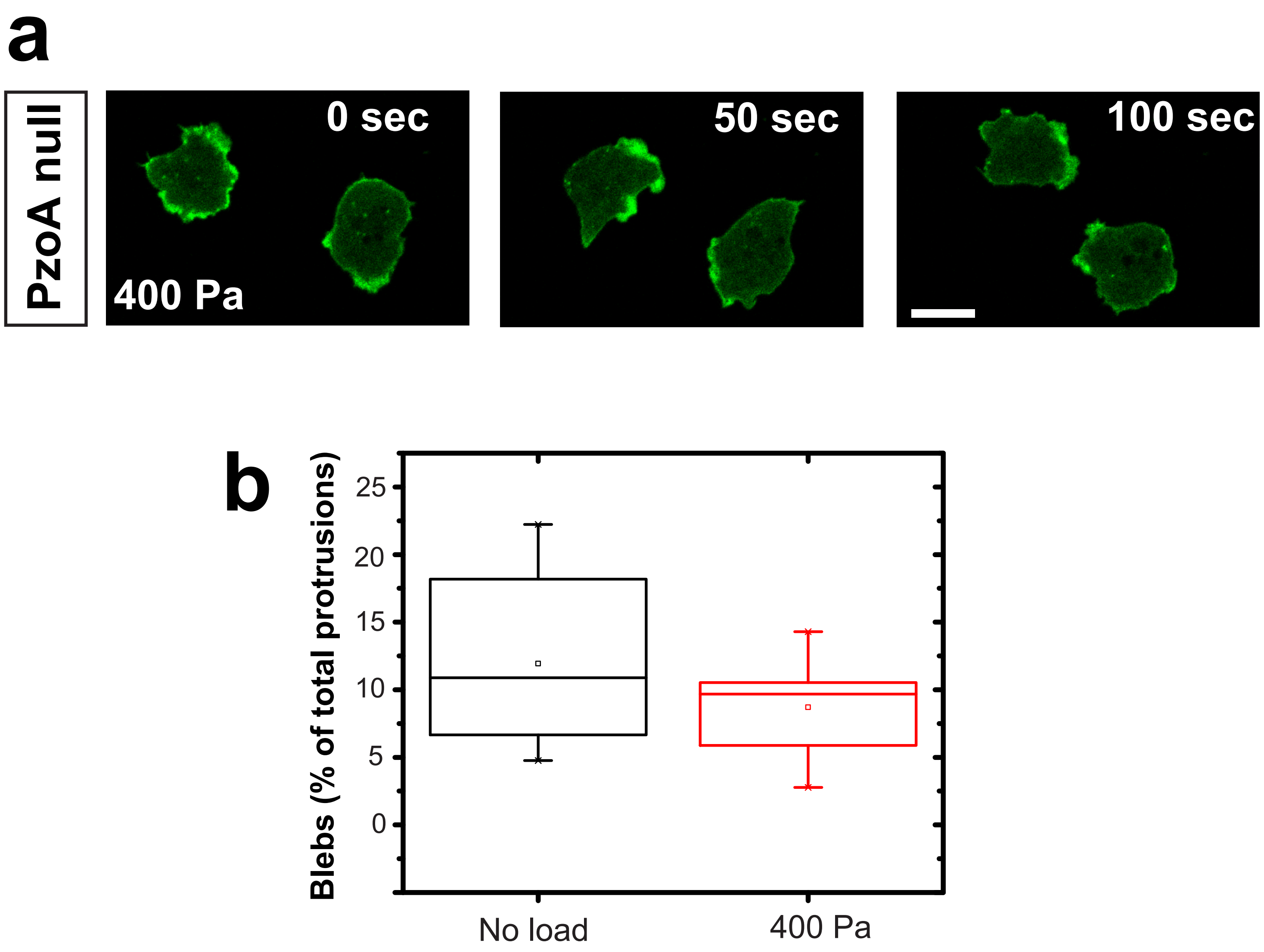
a
PzoA null
0 sec
400 Pa
50 sec
100 sec
b
Blebs (% of total protrusions)
25
20
15
10
5
0
No load
400 Pa

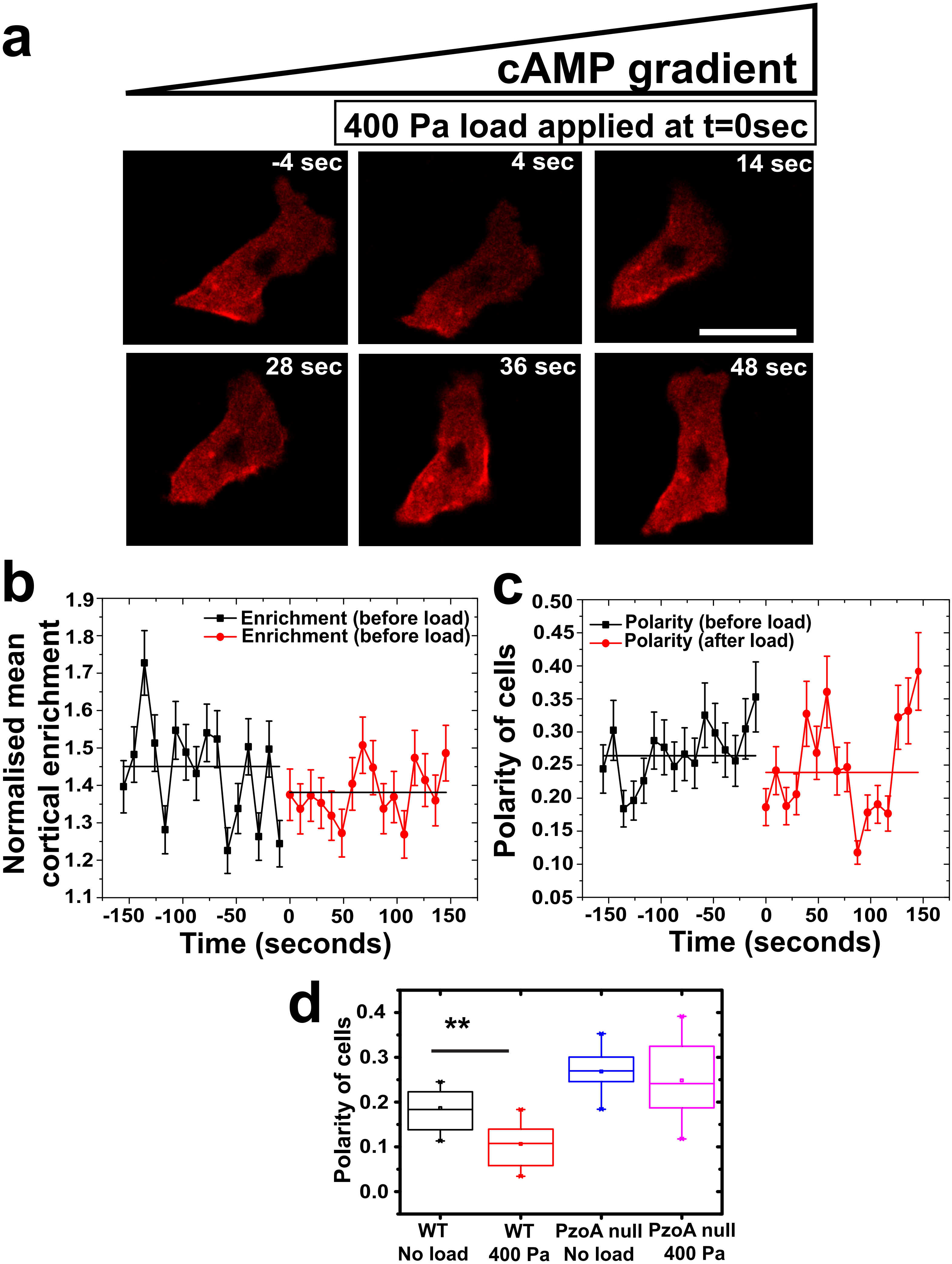
a
cAMP gradient
400 Pa load applied at t=0sec
-4 sec
4 sec
14 sec
28 sec
36 sec
48 sec
b
Normalised mean cortical enrichment
Enrichment (before load)
Enrichment (before load)
Time (seconds)
c
Polarity of cells
Polarity (before load)
Polarity (after load)
Time (seconds)
d
Polarity of cells
**
WT No load
WT 400 Pa
PzoA null No load
PzoA null 400 Pa
